# The developmental transcriptome of contrasting Arctic charr ( *Salvelinus alpinus*) morphs

**DOI:** 10.12688/f1000research.6402.2

**Published:** 2016-04-25

**Authors:** Johannes Gudbrandsson, Ehsan P. Ahi, Sigridur R. Franzdottir, Kalina H. Kapralova, Bjarni K. Kristjansson, S. Sophie Steinhaeuser, Valerie H. Maier, Isak M. Johannesson, Sigurdur S. Snorrason, Zophonias O. Jonsson, Arnar Palsson

**Affiliations:** 1 Institute of Life and Environmental Sciences, University of Iceland, Reykjavik, 101, Iceland; 2 Holar University College, Saudarkrokur, 551, Iceland

**Keywords:** Salmonids, Aquaculture, ecomorphs, Polymorphism, parallel evolution, immunology, craniofacial divergence, mtDNA

## Abstract

Species and populations with parallel evolution of specific traits can help illuminate how predictable adaptations and divergence are at the molecular and developmental level. Following the last glacial period, dwarfism and specialized bottom feeding morphology evolved rapidly in several landlocked Arctic charr *Salvelinus alpinus* populations in Iceland.

To study the genetic divergence between small benthic morphs and limnetic morphs, we conducted RNA-sequencing charr embryos at four stages in early development. We studied two stocks with contrasting morphologies: the small benthic (SB) charr from Lake Thingvallavatn and Holar aquaculture (AC) charr.

The data reveal significant differences in expression of several biological pathways during charr development. There was also an expression difference between SB- and AC-charr in genes involved in energy metabolism and blood coagulation genes. We confirmed differing expression of five genes in whole embryos with qPCR, including *lysozyme* and *natterin-like* which was previously identified as a fish-toxin of a lectin family that may be a putative immunopeptide. We also verified differential expression of 7 genes in the developing head that associated consistently with benthic v.s.limnetic morphology (studied in 4 morphs). Comparison of single nucleotide polymorphism (SNP) frequencies reveals extensive genetic differentiation between the SB and AC-charr (~1300 with more than 50% frequency difference). Curiously, three derived alleles in the otherwise conserved 12s and 16s mitochondrial ribosomal RNA genes are found in benthic charr.

The data implicate multiple genes and molecular pathways in divergence of small benthic charr and/or the response of aquaculture charr to domestication. Functional, genetic and population genetic studies on more freshwater and anadromous populations are needed to confirm the specific loci and mutations relating to specific ecological traits in Arctic charr.

## Introduction

Historical contingencies and chance shape organisms during evolution ^1, 2^, but convergence in phenotype and molecular systems indicates that evolution is to some extent predictable ^3, 4^. Identification of genes and variants that influence evolved differences is not a trivial task ^5^. Ideal systems to study the role of chance and necessity in ecological evolution would be related species or populations with readily observable phenotypic variation, living in a tractable ecological setting, and showing parallel evolution of specific traits within/among species/populations. Examples of such species complexes are provided finches of the Galapagos islands ^6^, while cichlids of the African great lakes also provide an exciting mulit-species system in the same respect ^7^. The threespine stickleback has also emerged as a model “single species” system ^8^. The amount of diversity in the feeding specializations of fish provide great opportunities for studying adaptation and divergence at the developmental and genetic level.

One approach to identify pathways related to function or morphological differences between species, populations or ecomorphs is to study gene expression during development ^9, 10^. For example a microarray study of liver samples from anadromous and resident populations of brown trout ( *Salmo trutta*), revealed that gene expression in juveniles was more influenced by life history than relatedness ^11^. Furthermore, Filteau *et al.* (2013) ^12^ found a set of coexpressed genes differenting two whitefish morphotypes, implicating Bone morphogenesis protein (BMP) signaling in the development of ecological differences in trophic morphology. Thus we were quite keen to apply RNA-sequencing to analyze ecomorphs in our study system, Arctic charr. Two previous studies have used RNA-seq to study salinity tolerance in adult Arctic charr, and found links between gene expression and quantitative trait loci ^13, 14^.

Some northern freshwater fish species exhibit frequent parallelism in trophic structures and life history and in several cases are found as distinct resource morphs ^8, 15– 19^. One of these species, Arctic charr ( *Salvelinus alpinus*), is well suited for studying the developmental underpinnings of trophic divergence and parallel evolution. Local adaptation has been extensively studied in the salmonid family, to which Arctic charr belongs ^20^. The family is estimated to be between 88–103 million years old ^21, 22^. A whole genome duplication event occurred before the radiation of the salmonid family ^21– 24^ which has provided time for divergence of ohnologous genes (paralogous genes originated by whole genome duplication event). Furthermore, recent estimates from the rainbow trout ( *Oncorhynchus mykiss*) genome suggest that ohnologous genes are lost at a rate of about 170 genes per million years, and that on the order of 4500 were retained in rainbow trout ^22^. *De novo* assembly of genomes and transcriptomes is complicated if many paralogs are present, which is the case in Arctic charr - see 13, 14. In this study we opted for mapping the reads (36 bp) to a related reference genome/transcriptome ^25^, instead of *de novo* assembly.

### Molecular studies of the highly polymorphic Arctic charr

**Figure 1.**
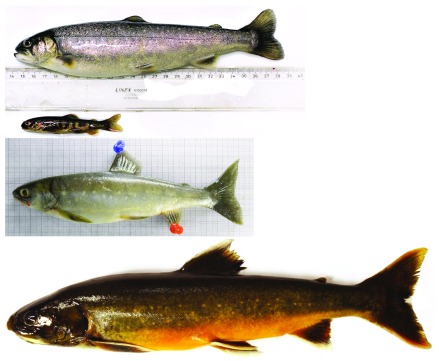
The Arctic charr morphs used in this study. Adult individuals of the four morphs studied here, from above; the Holar aquaculture charr, the small benthic charr; the planktivorous charr; and the large benthic charr. The latter three all come from Lake Thingvallavatn and were sexually ripe. The morphs differ in size at maturation, body and head shape - mainly lower jaw and length of maxilla and colour pattern in the wild.

Following the end of the last glacial period, about 10.000 years ago, Arctic charr colonized northern freshwater systems ^26^. It is found as anadromous or lake/stream residents and exhibits high level of within species polymorphism ^17, 26^. Charr is also known to harbour substantial phenotypic plasticity, which may promote or reduce divergence ^15, 27^. Resource polymorphism in charr correlates with ecological attributes ^28– 30^. For instance small charr with benthic morphology, are found in multiple lavaspring and pond habitats in Iceland ^31^, and a comparative study of Icelandic lakes ^30^ found that lakes with greater limnetic habitat, lower nutrients levels, and greater potential for zooplankton consumption appeared to promote resource polymorphism. Some of the larger lakes contain two or more distinct morphs, typically limnetic and benthic forms. Multiple lines of evidence show that these differences stem both from environmental and genetic causes ^32– 36^. The best studied example of sympatric charr are the four morphs in Lake Thingvallavatn ^37^; two have a benthic morphotype, a large benthivorous (LB-charr) and a small benthivorous (SB-charr), and two morphs are limnetic, a large piscivorous morph (PI-charr) and small planktivorous morph (PL-charr) ^38^. Both PL and PI-charr operate in open water and feed on free-swimming prey, PL on planktonic crustaceans and PI on small fish. The PL, LB and SB-charr are presented in Figure 1.

Several population genetics studies, using allozymes or mtDNA revealed no differences among charr morphs in Lake Thingvallavatn ^39– 41^ while other studies using microsatellite markers and nuclear genes, found significant ^42– 44^ genetic differences among morphs in the lake ^45^. Importantly Kapralova *et al.* (2011) ^44^ concluded that small benthic morphs have evolved repeatedly in Iceland and that gene flow has been reduced between the PL and SB morphs in Lake Thingvallavatn since its formation approximately 10,000 years ago ^46^. We also discovered genetic separation in immunological genes ( *MHCIIα* and *cath2*) between morphs in Iceland and within the lake ^45^, consistent with ecologically driven evolution of immune functions. Recently qPCR analyses showed that expression of mTOR pathway components in skeletal muscle correlates with the SB-charr form in Iceland ^47^, but it is unknown whether there is genetic differentiation in those genes or upstream regulators. Because individual genes have distinct histories ^48, 49^, genome wide methods are needed to identify genes and mutation that associate with divergence. Icelandic aquaculture charr (AC) was founded with fish from the north of Iceland, and has been bred at Holar University College since 1990 ^50^. The Holar AC-charr has responded to artificial selection in growth and performance characteristics, and is now the dominant charr breed in aquaculture in Iceland. While clearly a derived form, it has retained general limnetic craniofacial morphotype ( Figure 1).

In this study we compare SB-charr from Lake Thingvallavatn and AC-charr because i) SB charr represents an extensively studied and derived form of charr, that has been separated from Anadromous fish for approx. 10,000 years, ii) of the availability of abundant AC material and iii) we wanted an extreme contrast, because of budget reasons we could only sequence 8 samples at the time. The AC-charr is included here as a limnetic reference population, in part because we were unable to catch spawning anadromous charr, the ideal outgroup. But by focusing the follow up work on sympatric benthic and limnetic morphs of Lake Thingvallavatn, we can test and verify a subset of the signals found here. The contrast of SB and AC is justified as the data and studies ( 51– 53) building on this data illustrate (see discussion).

The overall objectives of our research program are to investigate the genetics and developmental underpinnings of charr divergence and benthic parallelism. As a step towards this we compare the developmental transcriptome of SB charr and AC charr. The aims of this study are threefold. First, to find genes and pathways related to the development of phenotypic differences between small benthic charr from Lake Thingvallavatn and Icelandic aquaculture charr conforming to a limnetic morphotype. Second, to screen for signals of genetic differentiation between these two charr types. Third, we set out to verify a subset of the expression and genetic signals, in these morphs and two more (benthic and limnetic) morphs from Lake Thingvallavatn. We conduct RNA-sequencing of developing offspring of these two contrasting Arctic charr morphs, reared in common lab environment to minimize the effects of environmentally induced phenotypic plasticity on developmental phenotypes and gene expression. The data reveal genetic changes in nuclear and mitochondrial genes and differential expression of genes that may affect craniofacial and phenotypic traits which separate benthic and limnetic morphotypes in charr.

## Methods

### Sampling, rearing and developmental series

**Figure 2.**
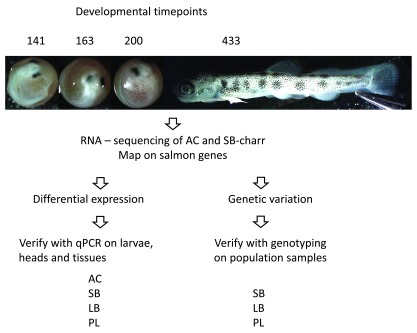
Schematic of RNA sequencing and follow up qPCR and population genetic work. RNA from embryos of the AC and SB charr at four stages (AC embryos pictured at top) were sequenced with Illumina technology. To verify differentially expressed genes we used RNA from embryos and heads of these four morphs, and tissues from adult AC charr. To verify SNPs we genotyped population samples from three Lake Thingvallavatn morphs (PL, LB and SB).

Overview of the experimental design, RNA sequencing, analyses and follow work is outlined in Figure 2. We set up crosses and reared embryos in the laboratory as described in 51. Embryos from four charr morphs were studied: an aquaculture charr (AC-charr) from the Holar breeding program ^50^ and three natural morphs from Lake Thingvallavatn; SB, LB and PL-charr ^54^. Samples of the first two, AC and SB-charr, with contrasting adult size and morphology ( Figure 1), were collected in 2009 and material sent for RNA sequencing. The latter two were sampled in 2010 and were used for qPCR and SNP studies of selected genes. Briefly, in September 2009 we got material from spawning AC-charr from the Holar breeding program, from single parent crosses ^50^ and spawning SB-charr collected via gill netting in Olafsdrattur in Lake Thingvallavatn. Similarly, in the 2010 spawning season SB-, LB- and PL-charr were collected from Lake Thingvallavatn. For each parent group, eggs from several females (3–10) were pooled and fertilized using milt from several males (3–5) from the same group. Embryos were reared at ~ 5°C under constant water flow and in complete darkness at the Holar University College experimental facilities in Verid, Saudárkrókur. The water temperature was recorded twice daily and the average was used to estimate the relative age of the embryos using tausomite units ( *τs*) ^55^. Embryos and juveniles were sampled at designated time points, placed in RNAlater (Ambion) and frozen at −20°C. Post hatching juveniles were reared at the same temperature on standard Aquaculture food. For the investigation of different tissues of adult aquaculture charr (AC) from Hólar (fish size 20–25 cm) were used. Six randomly selected individuals were killed (by cutting through spinal cord) and dissected, and samples were taken from the skin, heart, liver, gills, spleen, intestine and kidney of each fish. The samples were placed in RNAlater (Ambion) and stored at −20°C. We used DNA for population genetic analyses from our previous study ^45^, eight individuals from each of the three types, PL, LB and SB-charr.

Fishing in Lake Thingvallavatn was done with permissions obtained both from the owner of the land in Mjóanes and from the Thingvellir National Park commission. Ethics committee approval is not needed for regular or scientific fishing in Iceland (The Icelandic law on Animal protection, Law 15/1994, last updated with Law 157/2012). Sampling was performed by Holar University College Aquaculture Research Station (HUC-ARC) personnel. HUC-ARC has an operational license according to Icelandic law on aquaculture (Law 71/2008), which includes clauses of best practices for animal care and experiments.

### RNA extraction and transcriptome sequencing

Embryos of AC- and SB-charr sampled in 2009 were used for transcriptome sequencing. For this we focused on the time covering development of pharyngeal arches and morphogenesis of the head: at 141, 163, 200 and 433 *τs* (post fertilization). For each combination of morphs and timepoints we pooled RNA from approximately six individuals. RNA extraction and following steps were performed as described earlier ^51, 56^. Briefly, the embryos were dechorionated and homogenized with a disposable Pellet Pestle Cordless Motor tissue grinder (Kimble Kontes, Vineland, NJ, USA) and RNA was extracted into two size-fractions using the Ambion mirVana kit (Life Technologies, Carlsbad, CA, USA). The high molecular weight fraction was further used for mRNA-seq and RNA quality was analysed using an Agilent 2100 Bioanalyzer (Agilent Technologies, Santa Clara, CA, USA). RNA from samples was pooled - equal contribution of each sample - and first and second strand cDNA synthesis, fragmentation, adapter ligation and amplification were performed using the mRNA-Seq 8-Sample Prep Kit (Illumina, San Diego, CA, USA) according to manufacturer’s instructions. Sequencing was performed at DeCode genetics (Reykjavík, Iceland) using SOLEXA GAII technology (Illumina, San Diego, CA, USA).

The sequencing reads were deposited into the NCBI SRA archive under BioProject identifier PRJNA239766 and with accession numbers: SRX761559, SRX761571, SRX761575, SRX761577, SRX761451, SRX761461, SRX761490 and SRX761501.

The embryos sampled in 2010 were used for qPCR analyses. RNA was extracted from six whole embryos, in two replicates (two repetitions *X* three fish) (AC and SB sampled at 161 and 200 *τs*). For the extraction of RNA from heads of AC, SB, LB and PL, 12 embryos (two repetitions *X* six fish) at 178, 200 and 216 *τs* were used. Embryos were dechorionated and decapitated in front of the pectoral fin. RNA extraction and cDNA preparation were performed as described previously in 51. Similarly, RNA was extracted from a small piece (approximately 2 mm ^2^) of skin, heart, liver, gill, spleen, intestine and liver from six adult AC-charr.

### Analyses of RNA-seq data and mapping to Salmon EST contigs

As no *S. alpinus* genome is available and *de novo* assembly of the 36 bp reads yielded an excessive number of short contigs we chose to assess expression and genetic variation by mapping the reads to 59336 *S. salar* expressed sequence tag (EST) contigs from the SalmonDB [ 57, downloaded 22. March 2012] and the Arctic charr mitochondrial genome [ 48, NC_000861].

To estimate expression, reads were aligned with RSEM version 1.1.18 with default parameters. RSEM distributes reads that map to multiple locations to the most likely contig, using expectation maximization ^58^. The read counts for contigs with the same annotation were pooled because some genes were represented by more than one contig, and due to whole genome duplication almost the half of salmonid genes exist as ohnologs ^22, 24^. Thus the expression tests are done on gene or paralog group level, instead of the contig level. We acknowledge that paralogous genes are not always expressed similarly, but feel its necessary to do this pooling because of the nature of the data. In the remainder of the paper, we will refer to gene or paralog group (the number of underlying contigs is indicated in relevant tables). This brought the number of genes considered down to 16851. Lastly, paralog groups with fewer than 800 mapped reads in the entire dataset were excluded from the analyses, yielding a total of 10496.

A generalized linear model (GLM) with morph and developmental time as explanatory variables was used to find genes with different expression levels between the two charr morphotypes (groups) using the edgeR-package in R ^59^.

Y=Morph+Time+Error

To obtain further insight into the expression profiles of differently expressed genes, we performed clustering analyses on log-transformed cpm-values (counts per million; cpm-function in edgeR). The values for each gene were scaled by mean and standard deviation, and the euclidean distance used for the hclust-function in R ^60^ with the default settings. We used the hypergeometric-test in goseq ^61^ to test for gene ontology enrichment. Since we pooled the read-count from different contigs we could unfortunately not take gene length into account in those tests.

### Tests of differential expression with qPCR

We previously identified suitable reference genes to study Arctic charr development ^51^. Here we examined the expression of several genes in whole charr embryos, embryonic heads and adult tissues. Primers were designed using the Primer3 tool ^62^ and checked for self-annealing and heterodimers according to the MIQE guidelines ^63^ ( S1 Table). Primers for genes with several paralogs were designed for regions conserved among paralogs, except for *natterin-like,* where primers were designed to match regions differing in sequence between paralogs. Relative expression was calculated using the 2 ^−ΔΔ *Ct*^ method ^64^. For the calculation of relative expression of genes in whole embryos, the geometric mean expression of three reference genes, *β*- *Actin (Actb), elongation factor* 1 *α* and *Ubiquitin-conjugating enzyme E2 L3*, was used for normalization. For visual comparisons among samples, the normalized expression was presented as relative to the expression in AC at 161 *τs* (calibration sample). For the embryonic head samples *Eukaryotic Translation Initiation Factor 5A (If5a1)* and *Actb* were used as reference genes and a biological replicate of AC at 178 ( *τs*) as the calibrator sample, see 51, 52. Standard errors of relative expression were calculated from the standard errors (SE) of the Δ *C*_*T*_-values with the formula 2 ^−(ΔΔ *Ct*+ *SE*)^ = minimum fold expression and 2 ^−(ΔΔ *Ct*− *SE*)^ = maximum fold expression. The statistical analysis was performed using the Δ *C*_*T*_-values with a two-way ANOVA with GLM function in R.

Y=Morph+Time+MxT+Error

Normal distribution of residuals was confirmed for all data. For the study of expression in the embryonic head we followed a significant morph effect in the ANOVA with Tukey’s post-hoc honest significant difference test, on relative expression ratios (Δ *C*_*T*_s). Three genes had lower efficiency (as low as 1.72). We acknowledge that the data on those genes may be weak.

### Polymorphisms in the Arctic charr transcriptome

For analysis of genetic variation we mapped the reads to the salmon contigs, this time using the Burrow-Wheeler Aligner (BWA) ^65^ with a seed length of 25, allowing two mismatches. We re-mapped the reads, since BWA allows short indels (RSEM does not) but disregarding them leads to many false SNPs close to indels. To extract candidate polymorphic sites from the Arctic charr transcriptome we ran VarScan2 ^66^ with minimum coverage of 50 reads and minimum minor allele frequency of 0.1 on reads mapped to each *S. salar* contig for all of the 8 timepoints and morph combinations. This was done separately for reads that mapped uniquely to one contig only (UNI) and reads that mapped to two or more contigs (REP). These SNP-candidates were further processed in R ^60^, following established principles for variant calling ^67^. SNP-candidates at 90% frequency or higher in all samples were disregarded, as they reflect differences between Arctic charr and *S. salar* and are not the focus of this study. SNP-candidates with poor coverage in specific samples - i.e. coverage of five or fewer reads in three or four samples of each morph - were removed. As the SNP analysis was done on individual contigs, differences among paralogs appear in the data. To address this we use the fact that each sample is a pool of few individuals, thus true SNPs are unlikely to have the same frequency in all samples. However, variants reflecting differences between paralogs will have similar frequency all samples (assuming steady difference in their expression in all samples). We evaluated differences between samples with Fisher exact tests, and only SNPs significantly different between samples with a *p* < 0.05 (with no multiple testing correction) were retained. To compare morphs, read numbers were summed over the four samples from each morph. A conservative approach was taken by focusing on SNP-candidates that showed the largest differences in frequency between morphs (delta), without adjusting for multiple testing (Fisher exact test, *p* > 5%). SNP-candidates with the highest frequency difference (delta > 95%) were manually processed and redundant candidates removed. A similar approach was used to mine for polymorphisms in Arctic charr mtDNA (NC_000861), using *S. salar* mtDNA as the outgroup (NC_001960.1).

We wrote a python script to predict the impact of SNPs within the mRNA sequences. Polymorphisms were categorized according to their location (3’UTR, coding, 5’UTR), and those within the coding region into synonymous or non-synonymous.

### Verification of candidate SNPs

We chose 12 candidate SNPs for verification (see below). As the AC-charr is not a random breeding population, and because our interest is on differences between wild morphs, we took random samples of spawning SB, LB and PL-charr from Lake Thingvallavatn (8 per morph) from our earlier study ^45^. Using the same PCR and DNA sequencing approach we genotyped 12 candidate SNPs ( S2 Table). Briefly, we first compared the Salmon genome and ESTs [ 57, downloaded 22. March 2012] and short contigs from our preliminary assembly of the Arctic charr transcriptome. This allowed us to infer the placement of the putative polymorphism in the locus, and design paralog specific primers for PCR (less than 1 kb amplicons). MJ tetrad machine was used for PCR and the program was 5 min. at 95°C, followed by 35 cycles of 30 sec. at 52°C, 1 min. at 72°C, 30 sec. at 95°C, ending with 12°C while waiting on the human. Each individual was genotyped by first amplifying the region of interest using PCR, followed by ExoSAP (Affymetrix), direct sequencing (BigDye) and finally run on an Applied Biosystems 3500xL Genetic Analyzer (Hitachi). Raw data was base-called using the Sequencing Analysis Software v5.4 with KBTMBasecaller v1.41 (Applied Biosystems). Ab1 files were run through Phred and Phrap and imported to Consed for visual editing of ambiguous bases and putative polymorphisms, and for trimming primers. The FASTA files were aligned with ClustalW online [ 68, http://www.ebi.ac.uk/Tools/msa/clustalw2/] and manually inspected in Genedoc ^69^. All sequences where deposited to Genbank as popsets under the accession numbers KP019972-KP020026.

### Comparative genomic analyses of sequence polymorphisms

Two approaches were used for genomic comparisons of verified SNPs in the mitochondrial genome. Using the charr mtDNA sequence we performed both a BLAST search on salmon ESTs (May 2013) and retrieved multiZ alignments of vertebrates from the UCSC genome browser (in September 2013). This yielded several hundred sequences from related fish and other vertebrates. The list was reduced to 20 sequences for visualization, by keeping members of the major taxa but removing more closely related sequences, aligned with ClustalW and manually adjusted in Genedoc. The species and genome versions used are; Human ( *Homo sapiens*, hg19), Lamprey ( *Petromyzon marinus*, petMar1), Fugu ( *Takifugu rubripes*, fr2), Medaka ( *Oryzias latipes*, oryLat2), Stickleback ( *Gasterosteus aculeatus*, gasAcu1), Tetraodon ( *Tetraodon nigroviridis*, tetNig2), Zebrafish ( *Danio rerio*, danRer6). We also downloaded from NCBI the sequence of whole or partial mtDNA from several fish species; Brown trout ( *Salmo trutta*, JQ390057 and AF148843), Broad whitefish ( *Coregonus nasus*, JQ390058), Legless searsid ( *Platytroctes apus*, AP004107), Pacific menhaden ( *Ethmidium maculatum*, AP011602), Icefish ( *Salanx ariakensis*, AP006231 and HM151535), Chain pickerel ( *Esox niger*, AP013046) and Western Pacific roughy ( *Hoplostethus japonicus*, AP002938). The three mitochondrial variants (numbered by the *S. alpinus* mtDNA - NC_000861) are; m1829G>A (CCACGTTGTGAAACCAAC[G/A]TCCGAAGGTGGATTTAGCAGT), m3211T>C (CGTGCAGAAGCGGGCATAAG[T/C]ACATAAGACGAGAAGACCCT) and m3411C>T (CTCTAAGCACCAGAATTT[C/T]TGACCAAAAATGATCCGGC).

## Results

### RNA sequencing characteristics

Each sample yielded good quality data, with sequencing depth from 49 to 58 million (average: 55 million) reads. To quantify the expression levels, the reads were aligned to a salmon EST-assembly ^57^. Around 20% of the reads mapped uniquely to the EST data ( S3 Table). A further 30% mapped to two or more contigs, probably representing paralogous genes, recent duplications or repeat-like elements within transcribed regions. A substantial fraction of the RNA-sequencing reads did not map to the contigs from *S. salar*. Analyses of those reads require an Arctic charr genome sequence or transcriptome assembly from longer and paired end reads, currently underway in our laboratory.

### Differential expression during Arctic charr development

**Figure 3.**
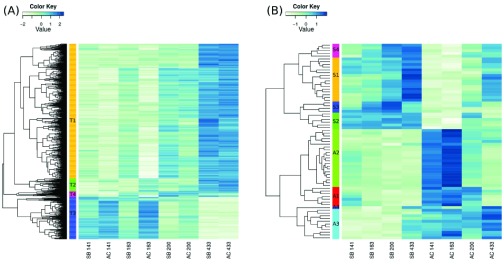
Heatmap of differentially expressed genes in the Arctic charr developmental transcriptome. Two morphs (SB and AC) are represented, at four timepoints. ( **A**) The 1603 genes with expression difference among time points, here clustered into four groups. ( **B**) The 71 genes differentially expressed between morphs are clustered into 4 groups for each morph. High expression is indicated by blue and low expression by beige.

We detected considerable changes in the transcriptome during Arctic charr development ( Figure 3a). The expression of 1603 and 2459 paralog groups differed significantly between developmental timepoints at the 1% and 5% levels of false discovery rate (FDR), respectively ( Dataset 1). The difference was most pronounced between prehatching (timepoints: 141, 163, 200 *τs*) and post hatching embryos (timepoint 433 *τs*), as more than 70% of the paralog groups with FDR below 1% had higher expression in the latter ( Figure 3b). Gene Ontology analyses reveal six enriched GO categories (below 10%FDR). The most drastic changes were seen in processes related to glycolysis (GO:0006096, FDR = 0.0009), where the expression of 19 out of 25 paralog groups changed during this developmental period. The other five classes that were differentially expressed during charr development are: ion transport (GO:0006811, FDR = 0.027), blood coagulation (GO:0007596, FDR = 0.03), DNA repair (GO:0006281, FDR = 0.08) and two immune related categories (GO:0019882, FDR = 0.08, GO:0006955, FDR = 0.09). Those results probably reflect developmental changes and/or differences in the environment of embryos before and after hatching.

### Differential expression between Arctic charr morphs

We were especially interested in genes showing expression differences between the two morphs as they might implicate pathways involved in the ecological divergence among charr populations. In the data 296 paralog groups were differentially expressed (FDR < 5%) between the morphs (141 higher in SB and 152 higher in AC-charr, Dataset 1). Among genes with higher expression in SB-charr two biological GO categories were enriched: blood coagulation (GO:0007596, p = 0.001) and proteolysis (GO:0006508, p = 0.002). Recall, expression of blood coagulation factors also differed between developmental stages (see above). In AC-charr, genes in three categories: respiratory electron transport chain (GO:0022904, p = 0.0006), ATP synthesis coupled electron transport (GO:0042773, p = 0.002) and neurotransmitter transport (GO:0006836, p = 0.009) have higher expression. The first two GO categories both relate to energy generation in mitochondria and could reflect higher expression of genes with mitochondrial functions in AC-charr. At more stringent FDR (1%), 31 paralog groups, with diverse functional annotations, were higher expressed in SB and 40 genes higher in AC-charr ( Figure 3b, Table 1 and Table 2). The higher expressed ESTs were clustered into 4 groups for each morph, reflecting in some cases functional similarity. For instance SB cluster 3 has three immune related paralog groups: *Complement factor D* (9), *H-2 class I histocompatibility antigen L-D alpha chain* (2) and *Sushi domain-containing protein 2* (4) ( Table 1). Note, however, that immune genes were not significantly enriched in the GO comparison of morphs. The results suggest genes with mitochondrial function, blood coagulation and other functions are differentially expressed between the morphs, but as few samples were sequenced, qPCR verification was needed.

### Validation of gene expression differences in whole embryos and paralog specific expression of *natterin* genes

**Figure 4.**
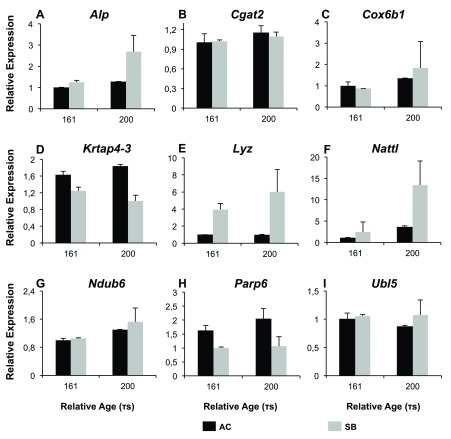
qPCR validation of candidates from transcriptome in whole embryos of Arctic charr. Relative expression of 9 genes ( **A**– **I**) analysed by qPCR in the small benthic (SB) charr from Lake Thingvallavatn and aquaculture (AC) charr at two different developmental timepoints (161 and 200 *τs*). 5 genes were differentially expressed between the two morphs ( *Alp, Krtap4-3, Lyz, Nattl, Parp6*), while 4 further genes did not show significant expression differences between morphs ( *Cgat2, Cox6B1, Ndub6, Ubl5*), see S4 Table. Error bars represent standard deviation calculated from two biological replicates.

For validation we opted for qPCR analyses of 9 genes/paralog groups in whole embryos and 8 in embryonic heads, which showed differential expression between AC and SB-charr, with statistical support ranging from <1% to about 10% FDR. We studied paralog groups with less FDR support, in part to be able to cast a wider net (see below). Of the nine paralog groups studied in whole embryos, five were confirmed to be differentially expressed between AC and SB-charr at 161 or 200 *τs* ( Figure 4, S4 Table and Dataset 2). Three genes, *Nattl, Alkaline phosphatase (Alp)* and *Lysozyme C II (Lyz2),* had significantly higher expression in SB. The other two, *Keratin-associated protein 4-3 (Krtap4-3)* and *Poly polymerase 6 (Parp6)* had higher expression in AC embryos ( Figure 4, S4 Table). No morph and time interaction was detected for any of the genes.

**Figure 5.**
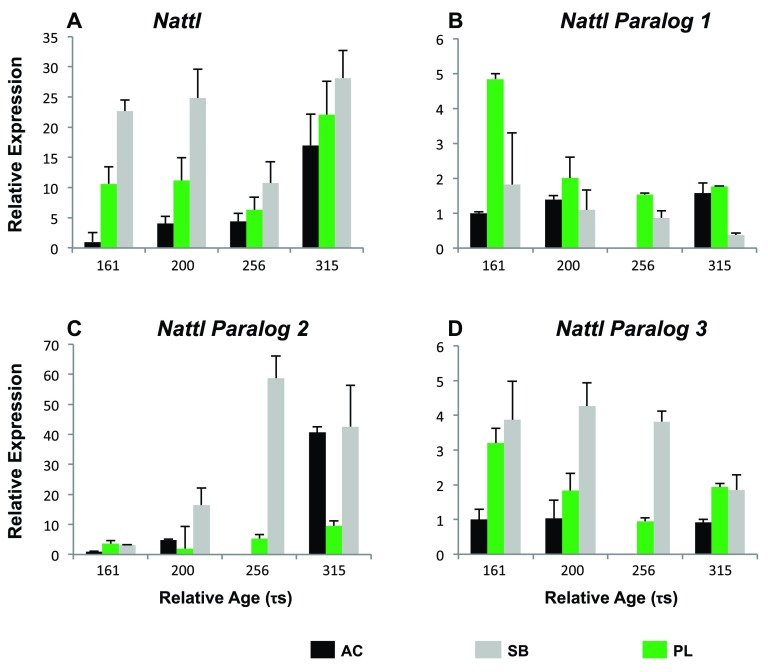
Relative expression of *Nattl* and its three paralogs during charr development in different morphs. The expression is graphed for different morphs (SB, AC and PL) at four developmental timepoints (161, 200, 256 & 315 *τs*, relative to AC-charr at timepoint 161. **A**) General *nattl* expression along charr development. **B–D**) Expression of *nattl* paralogs 1–3. ANOVA showing the variation among morphs is summarized in S5 Table.

As some genes are represented by different contigs or even paralogs, we set out to disentangle the expression of one paralog group ( *Nattl*) in detail. We measured the expression of three *natterin* paralogs ( *nattl1, nattl2 and nattl3*), by designing qPCR primers that matched divergent regions. These genes caught our interest because the only prior work implicated Natterin as a toxin produced by a tropical fish ^70, 71^. We studied *nattl* expression in several developmental stages in AC-, SB- and PL-charr as well as in selected tissues of adult AC-charr. The expression level of the three paralogs differed between morphs and timepoints ( Figure 5 and S5 Table). Overall *nattl2* had the highest expression in all morphs. The *nattl1* had higher expression in embryos of PL-charr than in AC- and SB-charr, while *nattl2* and *nattl3* were more expressed in SB-embryos. Note however, the efficiency of the primers for the *nattl* genes ranged from 1.72 to 1.77, which suggests this data should be interpreted with caution.

In order to evaluate the hypothesis that *nattl* genes have immune-related functions we studied expression in adult tissues (in AC-charr). The *nattl* expression was highest in the gills, followed by expression in kidney, skin and spleen. Low expression levels were detected in liver, intestine and heart ( S1 Figure and S5 Table). The three *nattl* paralogs followed different patterns, whilst each of them showed significant expression differences among tissues. *Nattl1* was mainly expressed in spleen and kidney, while *nattl2* showed a significantly higher expression in skin, liver and in gills. Similarly, the relative expression of *nattl3* was highest in the gills and skin. This indicates that the three *nattl* paralogs are expressed in a tissue specific manner, and also differently during the development of the three charr morphs studied here.

**Table 1.** Differentially expressed genes, with higher expression in the SB morph from Lake Thingvallavatn.

NR	Name	Abbr	Cont	logFC	logCPM	FDR	Cluster
3766	Histone H3 embryonic		1	8.71	2.74	7.80E-035	S-1
5103	Natterin-like	*Nattl*	6	2.75	7.12	7.76E-007	S-2
356	A7J6M9 Putative uncharacterized protein n175R		1	2.33	4.66	3.30E-006	S-1
6697	Q1KY05 Main olfactory receptor-like	*Sorf*	5	3.12	6.92	9.96E-005	S-1
8151	Sushi domain-containing protein 2	*Susd2*	4	2.20	5.55	0.0001	S-3
1682	Carcinoembryonic antigen-related cell adhesion molecule 1	*Ceacam1*	3	2.55	3.83	0.0002	S-1
6228	Protein FAM98A		2	1.96	4.76	0.0003	S-1
7531	STAM-binding protein-like	*Stampbl1*	2	2.07	2.62	0.0005	S-1
6712	Q1M160 Myc-regulated DEAD box protein		1	1.67	3.23	0.0009	S-1
2300	Cytosolic sulfotransferase 3	*Sult3st1*	3	1.73	2.13	0.0009	S-1
2063	Complement factor D	*Cfd*	7	1.79	6.42	0.0016	S-3
3326	Galectin-3-binding protein A		4	1.79	3.85	0.0017	S-4
3169	Flocculation protein 11	*Flo11*	2	1.86	4.05	0.0017	S-1
1203	B5XDY0 H-2 class I histocompatibility antigen L-D alpha chain		2	1.70	2.12	0.0028	S-3
9183	UPI000065D844 related cluster		2	1.97	5.55	0.0028	S-1
2909	Epidermis-type lipoxygenase 3	*Loxe3*	4	1.68	4.84	0.0029	S-1
4884	Myeloperoxidase	*Mpo*	4	2.20	6.78	0.0029	S-1
10003	Uridine phosphorylase 1	*Upp1*	4	1.51	3.00	0.0047	S-1
2513	Desmoglein-1-alpha	*Dsg1*	1	1.59	2.80	0.0054	S-2
377	A7SJA8 Predicted protein (Fragment)		1	1.73	2.50	0.0055	S-3
9204	UPI00006A2900 related cluster		2	6.38	3.26	0.0064	S-1
9642	UPI00017B1B0F related cluster		1	2.00	1.92	0.0064	S-2
1965	Coiled-coil domain-containing protein 136	*Ccdc136*	2	2.15	2.32	0.0064	S-2
9260	UPI0000F1D4BA PREDICTED		1	1.80	2.41	0.0065	S-2
738	Adseverin	*Scin*	8	1.58	5.51	0.0073	S-1
9678	UPI00017B4479 related cluster		1	2.18	1.97	0.0074	S-4
8339	Testin	*Tes*	4	1.50	4.93	0.0080	S-2
6840	Q4SNH3 Chromosome 8 SCAF14543		1	1.42	4.00	0.0080	S-1
1668	Carbohydrate sulfotransferase 6	*Chst7*	1	2.09	2.08	0.0090	S-4
8341	Testisin	*Prss21*	2	2.01	2.76	0.0090	S-4
6373	Protein asteroid homolog 1	*Aste1*	6	1.29	4.24	0.0090	S-4

Name: name of unigene or paralog group

Abbr: Abbreviated paralog group or gene name

Cont: Number of contigs

logFC: log Fold Change

logCPM: log Counts Per Million

FDR: False Discovery Rate

The cluster numbering corresponds to Figure 3.

**Table 2.** Differentially expressed genes, with higher expression in the AC morph.

NR	Name	Abbr	Cont	logFC	logCPM	FDR	Cluster
3465	Glutathione S-transferase P 1	*Gstp1*	1	-8.35	2.45	1.12E-019	A-2
2475	Dehydrogenase/reductase SDR family member 7	*Dhrs7*	2	-4.88	2.15	9.67E-014	A-3
6945	Q6NWE8 Sb:cb283 protein		3	-6.08	3.02	2.15E-013	A-2
399	A8DW32 Predicted protein		1	-5.32	6.38	4.27E-010	A-1
9682	UPI00017B4B48 related cluster		2	-3.70	2.81	2.61E-008	A-2
9817	Uncharacterized protein ART2		5	-12.63	6.89	8.23E-008	A-2
6724	Q2L0Z2 Putative ATP-dependent RNA helicase		1	-3.41	1.89	1.88E-007	A-2
1197	B5XD10 Vacuolar proton pump subunit G 1	*Atpv1g1*	1	-4.30	2.10	1.84E-006	A-2
5325	Nucleoside diphosphate kinase B	*Nme2*	1	-9.85	7.63	2.51E-006	A-1
9205	UPI0000D5B923: myelin basic protein isoform 1	*Mbpa*	3	-2.49	3.45	9.18E-006	A-3
6377	Protein broad-minded	*Tbc1d32*	1	-2.11	2.74	4.75E-005	A-1
5711	Pistil-specific extensin-like protein		1	-2.16	2.60	0.0002	A-3
3203	Formin-like protein 20	*Fmnl2b*	7	-1.98	1.95	0.0002	A-3
9315	UPI0000F2EC69: hypothetical protein		2	-5.60	4.57	0.0005	A-2
363	A7RFV0 Predicted protein (Fragment)		2	-1.74	4.96	0.0010	A-3
6937	Q6AZT1 MGC81677 protein		3	-2.06	3.81	0.0014	A-2
3756	Histone H1	*Histh1*	3	-2.26	4.54	0.0017	A-2
1133	B5DGN9 Creatine kinase-1	*Ckm1*	7	-4.72	5.50	0.0017	A-3
309	A1IMH7 CD80-like protein	*Cd80*	12	-1.94	4.29	0.0017	A-2
7651	Serine protease ami		2	-1.54	5.90	0.0017	A-3
9935	Uncharacterized protein C7orf63 homolog		1	-1.87	1.91	0.0025	A-2
5219	Nostrin	*Nostrin*	2	-2.55	3.38	0.0029	A-2
1855	Chondroitin sulfate N-acetylgalactosaminyl- transferase 2	* Csgalnact2*	5	-2.56	6.14	0.0034	A-1
10203	Xylose isomerase		6	-1.55	2.43	0.0035	A-3
2249	Cytochrome c oxidase subunit 3	*Cox3*	11	-1.78	11.15	0.0035	A-3
180	40S ribosomal protein S3-B	*Rps3b*	2	-5.31	8.67	0.0050	A-1
1227	B6NBL3 Putative uncharacterized protein		3	-1.59	2.95	0.0050	A-2
5055	NADH-ubiquinone oxidoreductase chain 6	*Nd6*	2	-1.48	2.65	0.0061	A-3
4634	Metallothionein A	*Mta*	1	-3.33	5.44	0.0064	A-2
342	A5C0J4 Putative uncharacterized protein		2	-2.58	2.47	0.0064	A-2
9698	UPI00019258B4: similar to epithelial cell transforming sequence 2 oncogene protein partial		1	-2.06	2.94	0.0064	A-2
5878	Pro-opiomelanocortin B	*Pomcb*	1	-2.04	5.60	0.0065	A-2
2248	Cytochrome c oxidase subunit 2	*Cox2*	9	-2.21	9.83	0.0074	A-2
1246	B8JI87 Novel protein similar to vertebrate collagen type VI alpha 3 (COL6A3) (Fragment)		1	-1.69	3.16	0.0080	A-3
7994	Sperm-associated antigen 5	*Spag5*	1	-2.07	3.71	0.0080	A-2
9515	UPI000175F90F: similar to pleckstrin homology domain containing family A member 7		1	-2.00	1.87	0.0090	A-2
1124	B5DDZ4 Acta1 protein	*Actc1b*	1	-1.52	2.62	0.0090	A-2
1127	B5DG94 2-peptidylprolyl isomerase A	*Ppia1*	2	-2.56	5.67	0.0090	A-1
9175	UPI000054A3C0 PREDICTED: apolipoprotein B		3	-1.32	4.09	0.0090	A-4
9671	UPI00017B3C62 related cluster		1	-1.51	1.92	0.0096	A-1

For column header explanation, see footer of Table 1.

### Expression differences in the developing heads of benthic and limnetic charr morphs

**Figure 6.**
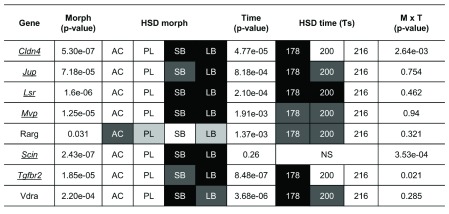
Expression differences of craniofacial candidate genes in developing head of Arctic charr morphs. Relative expression ratios, calculated from the qPCR data, were subjected to an ANOVA to test the expression differences amongst four charr groups and three close time points ( *τs*). The underlined gene names reflect significant difference between SB and AC-charr. A post hoc Tukey’s test (HSD) was performed to determine the effects of morphs, time and morph-time interaction (M X T). White boxes represent low expression, while black boxes represent high expression. The shading represents significant different expression between the samples (α = 0.05, NS = not significant). The genes studied were, *Claudin 4 (Cldn4)*, *adseverin (Scin)*, *Junction plakoglobin (Jup)*, *Lipolysis stimulated lipoprotein receptor (Lsr)*, *Major vault protein (Mvp)*, *Transforming growth factor beta receptor II (Tgfbr2) Vitamin D receptor a (Vdra)* and *Retinoic acid receptor gamma-A (Rarg)*.

To get a handle on the craniofacial divergence between sympatric Arctic charr morphs we used qPCR to study 8 paralog groups with expression difference in the RNA-seq data (all higher in SB). We focused on those with known craniofacial expression in zebrafish development ^72^ and compared two benthic (SB, LB) and two limnetic charr (AC, PL). We analyzed heads at three time-points (178, 200 and 218 *τs*) as this period overlaps with early stages of craniofacial skeletal formation in Arctic charr ^73, 74^. The qPCR confirmed the higher expression of seven out of these eight genes in the head of benthic charr compared to limnetic charr ( Figure 6, S2 Figure and Dataset 3). These seven genes are *Claudin 4 (Cldn4)*, *adseverin (Scin)*, *Junction plakoglobin (Jup)*, *Lipolysis stimulated lipoprotein receptor (Lsr)*, *Major vault protein (Mvp)*, *Transforming growth factor beta receptor II (Tgfbr2)* and *Vitamin D receptor a (Vdra)*. The eighth gene, *Retinoic acid receptor gamma-A (Rarg)* gave a small but significant response in the head, but the effects were reversed, i.e. the expression was higher in AC. The expression difference of the seven genes was, in almost all cases, consistent over the three timepoints studied (See S2 Figure). In summary the qPCR confirmed the differential expression of 12 of the 17 paralog groups studied ( Table 3), some which had 5–10% FDR support. To us that suggests substantial expression differences between these two charr morphs, and that the data can lead to hypotheses about morph specific activity in particular structures, like the developing head.

**Table 3.** Correspondence of transcriptome and qPCR verification on Arctic charr embryos.

Tissue	Name	Abbr	FDRm	FRDt	Effect	qPCR	Morph
Embryo	Alkaline phosphatase	*Alp*	0.070	0.001	0.986	*	SB
Embryo	Chondroitin sulfate N-acetylgalactosaminyltransferase 2	*Cgat*	0.004	0.331	-2.556		
Embryo	Cytochrome c oxidase subunit 6B1	*Cox6b1*	0.058	0.632	-1.208		
Embryo	B5X596 Keratin-associated protein 4-3	*Krtap4-3*	0.012	0.278	-1.986	*	AC
Embryo	Lysozyme C II	*Lyz2*	0.041	0.001	1.138	*	SB
Embryo	Natterin-like protein	*Nattl*	0.000	0.000	2.755	*	SB
Embryo	NADH dehydrogenase [ubiquinone] 1 beta subcomplex subunit 6	*Ndub6*	0.098	0.670	-1.175		
Embryo	Poly [ADP-ribose] polymerase 6	*Parp6*	0.108	0.379	-0.986	*	AC
Embryo	Ubiquitin-like protein 5	*Ubl5*	0.059	0.003	-1.234		
Head	Claudin-4	*Cldn4*	0.068	0.000	1.343	*	SB/LB
Head	Major vault protein	*Mvp*	0.065	0.528	0.958	*	SB/LB
Head	Junction plakoglobin	*Jup*	0.051	0.006	1.147	*	SB/LB
Head	Lipolysis-stimulated lipoprotein receptor	*Lsr*	0.013	0.043	1.369	*	SB/LB
Head	TGF-beta receptor type-2	*Tgfbr2*	0.065	0.013	1.728	*	SB/LB
Head	Vitamin D3 receptor A	*Vdra*	0.053	0.052	1.312	*	SB/LB
Head	Retinoic acid receptor gamma-A	*Rarg*	0.012	0.001	1.403		
Head	Adseverin	*Scin*	0.007	0.000	1.578	*	SB/LB

Tissue: which tissue was studied Abbr: abbreviated paralog group or gene name FRDm: FDR for comparison of SB and AC-charr in transcriptome FDRt: FDR for comparison among developmental timepoints in transcriptome Effect: logarithm of fold change between morphs, positive is higher in SB and negative higher in AC-charr in transcriptome (logFC.morph in supplemental dataset 1) qPCR: results consistent with transcriptome (*), a blank cell reflects lack of correspondence Morph: which morph(s) had higher expression in qPCR verification

### Analyses of polymorphism in Arctic charr transcriptome

The RNA-seq data also revealed segregating variations with large frequency differences between charr morphs. To uncover candidate SNPs we mapped the reads to all of the *S. salar* EST-contigs. Filtering on coverage yielded 165,790 candidate SNPs ( Table 4); of those 66.569 came from reads that mapped uniquely and 57.009 candidate SNPs from reads that mapped to more than one contig; with limited overlap between lists. Assuming that the expression of paralogous genes is stable, then differences among paralogs appear as SNPs at similar frequency in all samples. By requiring variant frequency differences (p < 0.05, uncorrected) between samples we reduced the list of candidates by two thirds, yielding over 20.000 candidate SNPs. Note, as cDNA from charr families was sequenced (not a population sample), estimates of SNP frequencies are imprecise. To err on the side of caution, we chose SNP candidates with 50% or higher frequency difference between morphs for further study. The candidate SNPs were also summarized by frequency of the derived allele, in reference to the *S. salar* sequence. This gave 672 and 872 SNPs at higher frequency, in AC-charr and SB-charr, respectively. The uniquely and multiply mapped reads, revealed approximately similar numbers of candidate SNPs. Gene ontology analysis showed that for derived SNPs in SB, there was an excess of variants in genes related to translation, both as a broad category and specific subgroups ( S6 Table). There was also enrichment of SNPs in genes related to DNA-mediated transposition, DNA integration, DNA replication and oxidation-reduction process. No GO categories were enriched for high frequency derived SNPs in AC. Furthermore, functional effects of the candidate SNPs (UTR, synonymous and non-synonymous) were predicted. The distribution among those categories did not differ between variants detected by uniquely or repeatedly mapped reads, $\chi_{\left[3\right]}^{2}=2.59$, *p* = 0.46 ( S7 Table).

**Table 4.** Candidate SNPs in the Arctic charr transcriptome, filtered by coverage, difference between sample and morphs and frequency difference between morphs.

SNP-candidates	Morph	Uni	Rep	Total
Total		96231	74341	165790
Filter coverage		66569	57009	113776
Diff. Bwn. samples		21417	22252	42869
Diff. Bwn. morphs		11385	12953	23974
Delta > 0.5	AC	396	285	672
Delta > 0.5	SB	526	353	872
Delta > 0.75	AC	95	68	159
Delta > 0.75	SB	155	95	248
Delta > 0.95 ^a^	AC	17	13	30
Delta > 0.95 ^a^	SB	29	4	33

SNP-candidates: found by mapping to *S. salar* ESTs

Uni/REP: from UNIquely or REPeatedly mapped RNA-reads

Delta: differences in allele frequency between morphs, categorized by which morph had the higher derived allele frequency

^a^The number of SNP-candidates before the redundant ones were removed

A total of 60 candidate SNPs are nearly fixed in one morph, with frequency difference between morphs above 95% (after manual inspection of contigs and SNP position three candidates were removed since they represented the same SNP). Of these “fixed” SNPs 46 came from uniquely mapped reads and 14 from reads that mapped more than twice ( Table 5 and Table 6). For the SNPs from uniquely mapped reads, 17 are fixed in AC-charr and 29 in SB-charr. The few genes with two or more polymorphic sites were; *Keratin type II cytoskeletal 3 (Krt3)*, *Cysteine sulfinic acid decarboxylase (Csad)* and *DNA-directed RNA polymerase I subunit RPA12 (Rpa12)* with 5, 5 and 2 SNPs respectively. *Krt3* and *Csad* had significant differentiation in both SB and AC. Similarly, 14 SNPs with large differentiation between morphs were predicted from reads that mapped on two or more contigs ( Table 6). Of these, we found two variants in the mitochondrial *60S ribosomal protein L36 (RpL36)* and variants in 4 other mitochondrial genes *(28S ribosomal protein S18a mitochondrial (MRPS18A)*, *Apoptosis-inducing factor 1 mitochondrial (AIFM1)*, *Isocitrate dehydrogenase [NADP] mitochondrial (acIDH1)* and *Protein S100-A1 (S100a1))*, all at higher frequency in AC-charr. PCR and Sanger sequencing of population samples confirmed SNPs in *DNA2-like helicase (Dna2)*, a gene with nuclear and mitochondrial function, and two other genes *Uroporphyrinogen decarboxylase (Urod)*, and *Mid1-interacting protein 1-like (Mid1ip1)* ( S2 Table). The candidate variant *Eukaryotic translation initiation factor 4 gamma 2 (Eif4g2)* was not substantiated by the PCR/sequencing.

**Table 5.** SNP candidates from uniquely mapped reads.

(a) Higher frequency in AC morph							
Contig	Annotation	Pos	Ref	Var	Freq-SB	Freq-AC	Effect
SS2U026955	Keratin type II cytoskeletal 3	300	A	T	0.000	0.984	synonymous
SS2U026955	Keratin type II cytoskeletal 3	309	G	A	0.000	0.996	synonymous
SS2U033960	Cysteine sulfinic acid decarboxylase	192	C	G	0.000	1.000	5prime
SS2U033960	Cysteine sulfinic acid decarboxylase	416	G	T	0.000	0.961	G to V
SS2U033960	Cysteine sulfinic acid decarboxylase	945	C	A	0.004	0.956	synonymous
SS2U043396	Eukaryotic translation initiation factor 2-alpha kinase 1	134	A	G	0.000	1.000	5prime
SS2U043886	Transcription cofactor HES-6	1308	T	C	0.000	1.000	5prime
SS2U044339	Intraflagellar transport protein 52 homolog	479	T	C	0.021	1.000	D to G
SS2U045168	Putative Peptide prediction	1275	G	A	0.000	1.000	3prime
SS2U045328	E3 ubiquitin-protein ligase DTX3L	388	G	A	0.000	0.977	synonymous
SS2U045990	Low-density lipoprotein receptor-related protein 1	135	T	C	0.000	0.969	synonymous
SS2U048125 ^a^	Transmembrane protein 131-like	480	G	A	0.000	1.000	synonymous
SS2U052747	Uridine 5’-monophosphate synthase	914	G	A	0.000	0.951	synonymous
SS2U054542	Mediator of RNA polymerase II transcription subunit 20	474	C	T	0.027	0.995	synonymous
SS2U056193	SUMO-conjugating enzyme UBC9	96	A	T	0.000	1.000	3prime
SS2U057101	ETS domain-containing protein Elk-3	440	C	G	0.000	1.000	3prime
SS2U058860	Voltage-dependent anion-selective channel protein 2	681	G	T	0.000	1.000	3prime
(b) Higher frequency in SB morph							
Contig	Annotation	Pos	Ref	Var	Freq-SB	Freq-AC	Effect
SS2U000399	Insulin-like growth factor-binding protein 7	598	C	A	1.000	0.000	3prime
SS2U004484	Titin	387	G	A	0.990	0.010	synonymous
SS2U026826	L-asparaginase	363	C	T	1.000	0.000	H to Y
SS2U026955	Keratin type II cytoskeletal 3	116	C	A	0.996	0.031	T to N
SS2U026955	Keratin type II cytoskeletal 3	264	C	T	0.970	0.008	synonymous
SS2U026955	Keratin type II cytoskeletal 3	317	C	T	1.000	0.002	T to M
SS2U033960	Cysteine sulfinic acid decarboxylase	363	C	T	1.000	0.025	5prime
SS2U033960	Cysteine sulfinic acid decarboxylase	387	C	T	1.000	0.030	synonymous
SS2U033960	Cysteine sulfinic acid decarboxylase	657	T	C	0.990	0.031	synonymous
SS2U034322	Cyclin-C	1094	A	G	1.000	0.000	3prime
SS2U034431	Dolichyl-diphosphooligosaccharide–protein glycosyltransferase subunit 2	436	G	A	0.992	0.000	G to S
SS2U036025	Nuclear receptor coactivator 4	36	G	A	1.000	0.043	5prime
SS2U040590	Glutamyl-tRNA(Gln) amidotransferase subunit A homolog	478	G	A	0.972	0.000	synonymous
SS2U045606	Superkiller viralicidic activity 2-like 2	500	C	T	1.000	0.000	synonymous
SS2U047816	Squalene synthase	1139	G	A	1.000	0.029	synonymous
SS2U048063	Lysine-specific demethylase NO66	669	C	T	1.000	0.000	synonymous
SS2U050394	UPF0542 protein C5orf43 homolog	596	G	A	1.000	0.000	synonymous
SS2U050880 ^a^	Transmembrane protein 131-like	901	C	T	1.000	0.000	A to V
SS2U052076	Eukaryotic translation initiation factor 3 subunit A	824	C	T	1.000	0.031	synonymous
SS2U053417	RNA polymerase-associated protein LEO1	454	G	A	1.000	0.049	synonymous
SS2U054333	Scaffold attachment factor B2	382	G	A	0.999	0.000	V to M
SS2U054705	Cell division protein kinase 4	122	A	G	0.971	0.000	3prime
SS2U054965	DNA-directed RNA polymerase I subunit RPA12	106	G	A	1.000	0.000	5prime
SS2U054965	DNA-directed RNA polymerase I subunit RPA12	411	T	G	1.000	0.000	synonymous
SS2U055120	Chromatin modification-related protein MEAF6	350	A	C	1.000	0.000	H to P
SS2U055153	Complexin-1	1191	C	A	1.000	0.031	3prime
SS2U057635	Mitogen-activated protein kinase 14B	1370	A	T	1.000	0.026	3prime
SS2U058169	Transmembrane protein 50A	1214	C	G	0.973	0.000	3prime
SS2U058802	Signal recognition particle 54 kDa protein	607	T	A	0.969	0.000	C to S

^a^Those genes are distinct paralogs

**Table 6.** SNP candidates with significant difference frequency between AC and SB morphs, from reads that mapped to two or more contigs.

Contig	Annotation	Pos	Ref	Var	Freq-SB	Freq-AC	Effect
SS2U004839	Actin alpha sarcomeric/cardiac	550	A	C	0.015	0.999	3prime
SS2U021298	28S ribosomal protein S18a mitochondrial	462	A	C	0.000	1.000	synonymous
SS2U041264	Apoptosis-inducing factor 1 mitochondrial	341	C	T	0.000	0.952	synonymous
SS2U054211 ^a^	Cytoplasmic dynein 1 intermediate chain 2	136	T	C	0.018	0.974	synonymous
SS2U054362 ^a^	Q08CA8 Dynein cytoplasmic 1 intermediate chain 2	945	A	G	0.000	1.000	synonymous
SS2U055923	Bystin	1623	A	C	0.000	0.983	3prime
SS2U058758	Protein S100-A1	253	C	T	0.000	0.984	synonymous
SS2U059000	Isocitrate dehydrogenase [NADP] mitochondrial	1654	T	C	0.000	0.975	3prime
SS2U059146	60S ribosomal protein L36	263	T	G	0.009	1.000	synonymous
SS2U059146	60S ribosomal protein L36	470	A	C	0.009	1.000	synonymous
SS2U036667	Heterogeneous nuclear ribonucleoprotein K	813	C	T	1.000	0.022	5prime
SS2U042873	RNA polymerase-associated protein LEO1	460	G	A	1.000	0.000	synonymous
SS2U058455	Adenylosuccinate lyase	1616	C	T	1.000	0.000	3prime
SS2U058906	Mid1-interacting protein 1-like	350	G	T	0.985	0.000	E to D

^a^Those genes are distinct paralogs

### Polymorphism and expression of Arctic charr mtDNA

Considering the enrichment of differentially expressed genes related to mitochondrial energy metabolism (above), and high frequency candidate SNPs in several genes with mitochondrial function in AC-charr we decided to study the mitochondrial transcriptome further. The charr studied here reflect metabolic extremes, the aquaculture charr was bred for growth while the small benthic morph is thought to have experienced natural selection for slow metabolism and retarded growth ^38, 75^. Although mRNA preparation protocols were used for generating cDNA for the RNA-sequencing, a substantial number of reads came from non-polyadenylated sequences. By mapping the reads to mtDNA sequence of Arctic charr we could estimate expression and infer polymorphism both in genes and intergenic regions. There was a clear difference in sequencing coverage, with more than twice as many reads mapped from the AC- compared to SB-charr (mean fold difference 2.27, Wilcoxon test, p < 0.0004). Note, as only two types of fish are compared, the polarity of expression divergence is unknown.

**Figure 7.**
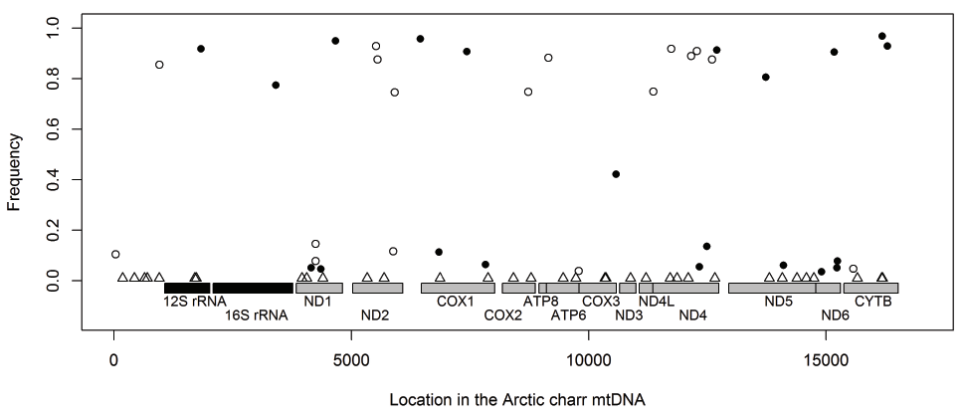
Genetic divergence in the mtDNA between SB- and AC-charr. The frequency differences between morphs of candidate SNPs, estimated from the RNA-sequencing, graphed along the mtDNA chromosome. The SNPs indicate whether the derived allele is of higher frequency in SB (black dots) or AC (open circles). Sites of divergence between the Icelandic stocks and the Canadian reference sequence are indicated by triangles. The two black boxes represent the rRNA genes and gray boxes the 14 coding sequences (abbreviated names underneath each gene).

**Figure 8.**
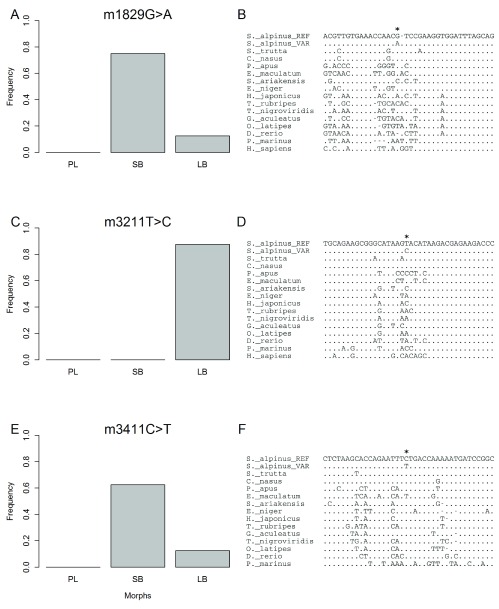
Comparative genomics and population genetic differentiation in Arctic charr at 3 mtDNA locations. Three variants in the 12s and 16s RNA genes are segregating in charr morphs in Lake Thingvallavatn. **A**, **C**, **E**) Frequency of each of those variants in three morphs from Lake Thingvallavatn (PL, LB and SB). A total of 8 individuals were genotyped from each morph, see methods. **B**, **D**, **F**) Aligned are several fish genomes, with Lamprey or humans as outgroups, reflecting a 38 bp window around each of the 3 positions (). Indicated are the two Arctic charr alleles, the reference allele (S._alpinus_REFcharr_WT) and the derived variant (S._alpinus_VARcharr_M). **B**) Alignment of variant m1829G>A in the 12s rRNA gene in fishes, using humans as an outgroup. **D**) Similar alignment of a 16s variant, m3211T>C and **F**) alignment of variant m3411C>T in the 16s rRNA gene.

The mapped RNA-reads were used to identify polymorphism and divergence in the entire mitochondrial chromosome. The polymorphisms were found by mapping to mtDNA from a Canadian *S. alpinus* ^48^, but ancestral vs. derived status inferred by comparison to *S. salar* mtDNA. This revealed 82 candidate sites, including 35 that represent divergence between Icelandic and Canadian charr. A total of 20 candidate SNPs had high (more than 50%) frequency difference between SB- and AC-charr ( Figure 7). There was no bias in the distribution of derived SNPs, 11 on the AC branch and 9 in SB. The divergence between Iceland and Canada is particularly little in the 12s and 16s ribosomal RNA genes. Curiously two SNPs in those genes differed strongly in frequency between morphs ( Figure 7). To confirm and better estimate the frequency of variants in the ribosomal genes, we PCR amplified and sequenced two ~550 bp regions in the rRNA genes, comparing three morphs (PL, LB and SB) from Lake Thingvallavatn ( Figure 8A, C & E, S2 Table). The 12s polymorphism (m1829G>A) differed significantly between the morphs ( $\chi_{\left[2\right]}^{2}=8.6$, *p* = 0.014), and was at highest frequency in the SB (0% in PL, 12.5% in LB and 75% in SB). Similarly m3411C>T in the 16s was enriched in SB (62.5%) but found at lower frequency in PL (0%) and LB (12.5%) (it differed significantly between morphs, $\chi_{\left[2\right]}^{2}=9.3333$, *p* = 0.009). The Sanger sequencing also revealed three other polymorphisms in the amplified region, not seen in the transcriptome. Among those m3211T>C in the 16s gene was at 75% frequency in LB, but not found in the other morphs ( $\chi_{\left[2\right]}^{2}=19.76$, *p* < 0.0001).

In order to gauge the potential functionality of those variants we aligned the rRNA genes from nearly hundred fishes and several vertebrates. The position affected by m1829G>A and m3211T>C, in the 12s and 16s rRNAs, are not well conserved in fishes or vertebrates ( Figure 8B & D). However m3411C>T, in the 16s rRNA, alters a position that is nearly invariant in 100 fish genomes ( Figure 8F). The only exception is Pacific menhaden, which curiously also has T in this position. This region could not be aligned properly in other vertebrates. Thus m3411C>T alters a conserved position, but probably not very drastically as the introduced allele is tolerated in another fish species.

Parameters and multiple testing corrected p-values for expression analysis The file is tab-delimited and the columns are; “Unigene.Description”: the annotation for that gene/paralog group. “NR.contigs”: number of contigs with this annotation. “logCPM”: count per million, log-scale. "logFC.morph": Mean fold change between the morphs, log-scale. "logFC.T163", "logFC.T200", "logFC.T433": Mean fold change for each timepoints compared to timepoint 141, log-scale. "FDR.morph": P-value for morph difference, multiple testing corrected. "FDR.time": P-value for time differences, multiple testing corrected. "Contigs": SalmonDB id for the contigs with the specific annotation ^109^. Click here for additional data file. Copyright: © 2016 Gudbrandsson J et al. 2016 Data associated with the article are available under the terms of the Creative Commons Zero "No rights reserved" data waiver (CC0 1.0 Public domain dedication).

qPCR data for tests of expression in charr developing embryos and adult tissues “Gene Type": Designates the reference and candidate genes. “Gene”: Name of the gene. “Morph”: Which charr type the sample came from. "Relative age": Developmental timepoint, and also indicates the samples from adult fish. "Biological replicate": The two or more biological replicates used. "cDNA No": Marks the cDNA isolation used. "Ct value": Estimate of gene expression. "Sample": Indicates the material used, whole embryos or distinct tissues. "Batch": Demarcates distinct collections of cDNA, applies only to nattl ^110^. Click here for additional data file. Copyright: © 2016 Gudbrandsson J et al. 2016 Data associated with the article are available under the terms of the Creative Commons Zero "No rights reserved" data waiver (CC0 1.0 Public domain dedication).

qPCR data for tests of expression in charr developing embryo heads “Gene Type": Designates the reference and candidate genes. “Gene”: Name of the gene. “Morph”: Which charr type the sample came from. "Relative age": Developmental timepoint. "Biological replicate": The two or more biological replicates used. "cDNA No": Marks the cDNA isolation used. "Ct value": Estimate of gene expression. "Tissue": Indicates the material used ^111^. Click here for additional data file. Copyright: © 2016 Gudbrandsson J et al. 2016 Data associated with the article are available under the terms of the Creative Commons Zero "No rights reserved" data waiver (CC0 1.0 Public domain dedication).

## Discussion

We are interested in the predictability of evolution at the molecular level, especially whether there exist principles that influence the rewiring of developmental and regulatory systems ^4, 76^. One way to study this is to identify genetic and developmental effects affecting key traits in species or populations which exhibit parallel evolution. The objective of this study were to get generate hypotheses about the genetic and molecular systems that associate with benthic morphology in charr by mainly focusing on the small benthic morph in Lake Thingvallavatn, Iceland. But as transcriptome were sequenced from embryos of SB-charr and aquaculture charr the data also reflect on the genetics of charr domestication.

### Developmental transcriptome of Arctic charr morphs

As no reference genome is available for Arctic charr, we mapped reads to *S. salar* EST-contigs ^57^ in order to estimate expression and identify candidate genetic polymorphisms. As many of the contigs are short or have overlapping annotations, we collapsed genes into paralogous genes when appropriate for the expression analysis. The main advantage of this approach was the reduction of the number of statistical tests (and hence an increase in statistical power). The downside is that paralog-specific expression patterns are masked, as our qPCR results of the *natterin like* gene family show ( Figure 5 and S1 Figure). Recent rainbow trout data shows about 1/4 of paralogs from the latest whole genome duplication event retain the very similar expression patterns ^22^ indicating that distinct expression patterns of two paralogs is quite common ^77^. In their analysis of the Arctic charr gill transcriptome, Norman *et al.* (2014) ^13, 14^ also used Illumina sequencing technology to evaluate expression. Their reads were longer (2x100 bp) than in this study (36 bp) enabling them to assemble contigs. They did not consider the paralogs in their approach and merged contigs based on sequence identity. Thus the complexity of Arctic charr transcriptome still remains unsolved. Our data reflects differential deployment of several gene classes during Arctic charr development. Studies in salmonids and other fish have demonstrated large changes in expression during early development, including coordinated changes in many cellular and developmental systems ^9, 78– 81^. Several blood coagulation factors genes showed significant changes during charr development, and were also more highly expressed in the SB-charr. This might reflect differences in the rate of development of blood composition, or tissue composition, in the two morphs. While our main interest is on the derived and repeatedly evolved small benthic charr, the data can also reflect differences due to domestication. In this study we chose to compare SB to AC-charr for several reasons, i) AC-charr has limnetic like head morphology, ii) was available for harvesting of running fish, and iii) because we wanted a strong contrast in this first survey of charr developmental diversity. The AC-charr proved a useful, as the data presented here has already revealed differential expression of several developmental genes and regulators with differential expression between benthic and limnetic charr ^51, 52^. Previously we found tight correlation of RNA-seq expression and qPCR estimates - using data from this very transcriptome ^51^. Furthermore, we have actually used the same morphs (AC and SB) and samples in a comparison of the developmental miRNA transcriptome – which reveal that expression of several miRNAs correlates with morph differences ^56^.

### Higher expression of *lysozyme II C* and *natterin-like* in SB-charr

Natural selection can shape variation in immunological genes. We decided to study further *Lyz2* and the putative immunological genes *nattl* that had higher expression in SB. The substrate of lysozyme ^82^ is the bacterial cell wall peptidoglycan and it acts directly on Gram-positive bacteria ^83^. Lysozyme also promotes the degradation of the outer membrane and therefore indirectly acts also on Gram-negative bacteria ^84^. Another gene that caught our attention was *natterin-like*. Natterins were first discovered from the venom gland of the tropical toxic fish species *Thalassophryne nattereri* ^70, 71^, and are found by sequence similarity in e.g. zebrafish, Atlantic salmon and here in Arctic charr. The Natterin proteins contain a mannose-binding lectin-like domain (Jacalin-domain). Mannose-binding lectins are pathogen recognition proteins (antibodies) and therefore are important for the acute phase response of fish ^85, 86^, thus we hypothesized that *nattl* genes in charr may have immune related functions. The data are consistent with this as the highest expression was found in skin and kidney. This putative immune functions needs to be verified. It is possible that higher expression of those two genes in SB-charr reflect preparation of juveniles for bottom dwelling habitats, which may be rich in bacteria and challenging for immune systems. One can ask whether immunological genes are expected to show similar or less parallelism than others genes shaped by natural selection? The current data does not reflect on this question, but our population genetic work shows genetic variation in immunological genes ( *MHCIIα* and *cath2*) does not correlate with the SB-charr ecotype in Iceland ^45^.

In this study we collapsed contigs into paralog groups for the transcriptome analyses. The disadvantage of this approach is that differential expression of a paralog, can be masked by related genes that do not differ between groups. We looked at this by studying the expression of three paralogs of the *natterin like* genes in different morphs during Arctic charr development, and among tissues of adult AC-charr. The data suggest that the three *nattl* genes are expressed differentially between the morphs, thus it is not divergence in the expression of one paralog that explains the general *nattl* expression disparity in the transcriptome. Certainly, other scenarios could apply to other genes in the transcriptome.

### Expression divergence in craniofacial genes in benthic morphs

A study of the skulls of charr post-hatching embryos and juveniles from Lake Thingvallavatn, showed that some elements of the developing head ossified earlier in SB-charr than in PL-charr ^87^. Morphometric analyses of developing heads (same stages as studied here) demonstrate differences in craniofacial elements between AC- and SB-charr, along a limnetic vs. benthic axis ^74^. Based on those developmental phenotypes we investigated further genes with roles in craniofacial development that were differentially expressed in the transcriptome. Guided by this transcriptome we had already found two extra-cellular matrix (ECM) remodeling genes, *Mmp2* and *Sparc* and a conserved co-expression module of genes with known roles in craniofacial morphogenesis, to have higher expression in developing heads of benthic Arctic charr morphs than in limnetic morphs ^51, 52^. Bioinformatic and qPCR analyses suggest the co-expression module may potentially be affected by quantity of the transcription factor *ETS2*. These studies and the current data confirm the utility of the contrasting developmental transcriptomes for identifying candidate genes with differential expression during head development, as 7 out of 8 candidates were confirmed by qPCR. These genes had consistently higher expression in the developing head of two benthic morphs (SB and LB), and lower in more limnetic fish (AC and PL). The most noteworthy aspect is the fact that three of the morphs (SB, LB and PL) are closely related and live in sympatry in Lake Thingvallavatn ^44^.

We focused on a few targets of Tgf- *β* and Ahr signaling pathways because of their role in craniofacial morphogenesis and transcriptional connection ^88– 90^. *Adseverin (Scin)* was one of the top differentially expressed genes ( Table 1) and has roles in rearrangements of the actin cytoskeleton, chondrocyte differentiation and skeletal formation ^91, 92^. Also, in the transcriptome *Lsr*, *Cldn4* and *Tgfbr2* had higher expression in SB-charr, and we show that higher expression of those genes associated with the benthic morphotype. *Lsr* is a molecular component of tri-cellular tight junctions ^93^ and has been shown to be suppressed upon Tgf- *β*1 stimulation ^94^ in a human cell line. Similarly, *Cldn4*, a tight junction protein with unknown role during embryonic morphogenesis, is a target of the Tgf- *β* and Ahr signaling pathways ^95, 96^. Finally, the expression of *Tgfbr2*, encoding a receptor of Tgf- *β* was slightly but significantly higher in the head of benthic morphs. Previous studies suggest a crucial role of *Tgfbr2* in craniofacial morphogenesis ^97^.

We also confirmed differential expression of other genes, including two with higher expression in SB-charr. *Mvp* is the predominant component of cytoplasmic ribonucleoprotein structures called vaults ^98^, which is highly conserved across eukaryotes. The vaults have been something of an enigma, but are implicated in several processes from signal transmission and immune response ^99^. The *Jup* gene also showed higher expression in SB-charr. Finally, higher expression of *Vdra*, encoding the vitamin D receptor A, was found in the heads of benthic charr. The receptor regulates mineral homeostasis, osteoblast differentiation and bone metabolism ^100^. A related study from our group, also building on this dataset, mapped in more detail the differential expression of these and other coexpressed genes in limnetic and benthic charr ^53^.

To summarize, the results show that RNA-sequencing of Aquaculture charr with limnetic craniofacial morphology and small benthic charr can be used to reveal differential expression of genes that associate with limnetic and benthic divergence in craniofacial elements in sympatric charr morphs. It would be interesting if expression of these genes associates with benthic morphology in independently evolved charr populations, as was seen for certain mTOR-pathway genes in muscle of adult SB-charr ^47^, or even in other species with similar trophic diversity.

### Genetics differences between the AC and SB-morphs - possibly in mtDNA function

Previous studies on microsatellite markers documented the population history of charr in Iceland and in particular the parallel evolution of SB-charr ^44^. Our data confirm genetic differences between SB and AC-charr. By comparing AC and SB-charr, that represents a small benthic resource morph that has evolved repeatedly in Icelandic stream and pond habitats ^44^, we hoped to implicate genes and pathways involved in adaptation to these special habitats. But the AC-charr is also interesting, as domestication over several decades has led to rapid growth and increased size ^50^. Morphometrics have not been used to compare the body or craniofacial shape of AC to other charr morphs, but domestication of *O. mykiss* has affected body shape and fin structure in partiuclar ^101^. The allele frequency differences and expression divergence observed can reflect neutral population genetic processes and/or selection during domestication or adaptation of SB-charr. By studying expression and allele frequencies in limnetic and benthic morphs from more locations, it may be possible to disentangle these questions. We restricted ourselves to verification of several SNPs, and focused mostly on variants in mtDNA because to us the data suggest interesting divergence in systems related to energy metabolism. First, there is 2X higher expression of respiratory electron transport chain components in AC compared to SB-charr and 100% more mitochondrial derived reads are found in the AC-charr samples. Note that the direction of divergence is unknown, i.e. whether expression was up in AC or down in SB. Second, many derived candidate-SNPs in genes related to mitochondrial function were at high frequency on the AC branch. For instance in *S100A1*, which has been implicated in mitochondrial regulation in cardiac tissue in humans ^102^, but its expression is probably not exclusive to this tissue. Third, while the mitochondrial ribosomal genes generally evolve slowly, we do see derived variants at high frequency in the SB and large benthic charr in Lake Thingvallavatn. Specifically, m3411C>T in SB affects a position that is highly conserved among fish, and could affect function of the 16s rRNA. Earlier studies of mitochondrial markers in *S. alpinus* did not find large signals of divergence within Iceland ^40, 42, 45^, probably because they studied other genes.

The mitochondrion is more than a powerhouse, it integrates metabolism, cell cycle and apoptosis ^103^. The number of mitochondria and its functions are known to correlate with environmental attributes. For instance in Antarctic fishes under extreme cold, higher numbers of mitochondria are found in muscle and heart cells ^104^. Our data suggest an expression difference between morphs that could reflect differences in total number of mitochondrion, the number of mtDNA copies per mitochondrion or cell, or difference in RNA expression from the mtDNA, possibly due to evolution of mtDNA related to diet and/or temperature ^105^. In sum, the results suggest divergence (adaptive or neutral) in mitochondrial function, due to the domestication of aquaculture charr and/or adaptation of the small benthic charr to its habitat in Lake Thingvallavatn. But further work is needed to map out the expression differences of mitochondrial related genes in more SB and anadromous charr morphs (representing the ancestral state). The mtDNA signals could also be investigated in populations along ecological clines (e.g. temperature) or with respect to life history ^106^.

## Conclusions

The data presented here suggest genetic and expression changes in multiple systems associate with divergence among the highly polymorphic and rapidly evolving Arctic charr in Iceland. The data reveal differential expression of two immunological genes between morphs and of several craniofacial developmental genes, that may help sculpture benthic vs. limnetic heads. The genetic data suggest among other things differentiation in the charr mtDNA between the SB and AC-charr morphs. It must be acknowledged that it is not trivial to identify genes affecting variation in ecologically important phenotypes, like shape ^107, 108^. Our broad interest is in how natural selection tweaks genetic regulatory systems, for instance via genetic changes in regulatory sequences or post transcriptional modifiers relating to adaptations. Genetic changes affecting gene expression can be raw material for adaptation, but could also rise in frequency due to reverberations in regulatory cascades ^76^. Following this work we plan to study the degree of developmental and population genetics parallelism of the small benthic charr, typically found in cold springs and small pond habitats in Iceland with lava substratum ^29, 44^. The availability of charr populations at different stages of divergence sets the stage for future genomic studies of the roles of genes, environment and plasticity for shaping this polymorphic species.

## Data availability

The data referenced by this article are under copyright with the following copyright statement: Copyright: © 2016 Gudbrandsson J et al.

Data associated with the article are available under the terms of the Creative Commons Zero "No rights reserved" data waiver (CC0 1.0 Public domain dedication). 

The sequencing reads were deposited into the NCBI SRA archive under BioProject identifier PRJNA239766 and with accession numbers: SRX761559, SRX761571, SRX761575, SRX761577, SRX761451, SRX761461, SRX761490 and SRX761501.

All DNA sequences where deposited to Genbank as popsets under the accession numbers KP019972-KP020026.

*F1000Research*: Dataset 1. Parameters and multiple testing corrected p-values for expression analysis, 10.5256/f1000research.6402.d48005 ^109^

*F1000Research*: Dataset 2. qPCR data for tests of expression in charr developing embryos and adult tissues., 10.5256/f1000research.6402.d48006 ^110^

*F1000Research*: Dataset 3. qPCR data for tests of expression in charr developing embryo heads., 10.5256/f1000research.6402.d48007 ^111^

## Figures and Tables

**S1 Figure.**
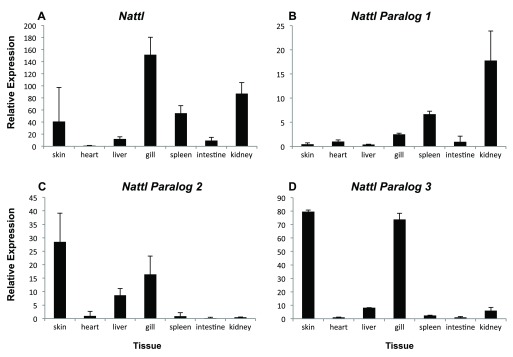
Relative expression of *nattl* and *nattl 1–3* in tissues of adult AC-charr. Relative expression of *Natterin* ( **A**) & *Natterin paralogs 1–3* ( **B**– **D**) within different tissues (skin, heart, liver, gill, spleen, intestine & kidney) of adult aquaculture charr (RT-qPCR); expression plotted for different tissues, relative to heart tissue (lowest expression levels).

**S2 Figure.**
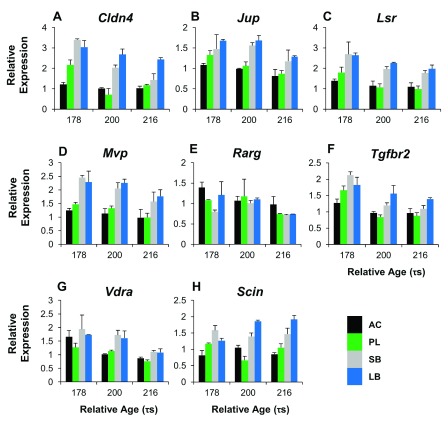
Relative expression of selected craniofacial candidate genes. Relative expression of 12 candidate genes with characterized craniofacial expression during zebrafish development (ZFIN website) in the head of SB, LB, PL and AC at three time points in development. In the transcriptome data all of the genes had shown higher expression in SB at 200 *τ*s. The expression is normalized to the geometric means of two craniofacial reference genes ( *ACTB* and *IF5A1*). Expression is relative to a replicate of AC morph at 200 (τs), set to one. Error bars represent standard deviation calculated from two biological replicates and each biological replicate contains homogenate of six heads.

**Supplemental Table S1 A.** qPCR primers used in this study.

Gene	Description	Primer Sequence (5’- 3’)	Product Size (bp)	PCR Efficiency	Melting Temperature (°C)	Exon Boundary
*Actb*	Beta Cytoskeletal Actin	F-GAAGATCAAGATCATCGCCC R-CAGACTCGTCGTACTCCTGCT	122	1.95	80.5 ± 0.7	Yes
*Alp*	Alkaline phosphatase	F-ACAGCATACCTCTGTGGGG R-GGTGGCATGGTTCACACG	177	1.90	85.12 ± 0.5	Yes
*Cldn4*	Claudin-4	F-GTGCTGTGC CATCCCAAG R-CACCACACAGGTCATCCACA	100	1.98	80.4 ± 0.6	Yes
*Cgat2*	Chondroitin beta-1,4-N- acetylgalactosaminyltransferase 2	F-GAGAGCCACTTTACTGAGGGG R-GAATGGACGGAAAAGAGTAACG	120	1.98	81.86 ± 0.3	Yes
*Cox6b1*	Cytochrome c oxidase subunit VIb isoform 1	F-GAGGGTCTACAAATCACTGTGC R-CCTGGAGTCCTACTCATACAAACAT	147	1.93	82.22 ± 0.7	Yes
*Ef1α*	Eukaryotic Translation Elongation Factor 1 Alpha	F-GAAGATCGGCTATAACCCTGC R-ACCTTCCATCCCTTGAACC	111	1.94	81.36 ± 0.4	Yes
*If5a1*	Eukaryotic Translation Initiation Factor 5A	F-GGCTTCGTGGTGCTGAAG R-CCATGTGGACCTTAGCGTG	91	1.91	80.76 ± 0.6	Yes
*Jup*	Junction plakoglobin	F-CACAGCAGACATACCAGGATG G R-CTGGCGATCTCTCCCCTGTT	109	1.97	81.0 ± 0.3	Yes
*Krtap4–3*	Keratin-associated protein 4–3	F-GCGGGACATCTACACTGCTTA R-AGAAGGCTAAAGTCTTAGTGACTATC	151	1.89	81.88 ± 0.6	Yes
*Lsr*	Lipolysis-stimulated lipoprotein receptor	F-TGCTGTCACTCTGGGCGA R-CCGTCTGGGCAAGGTTCA G	80	1.91	80.77 ± 0.5	Yes
*Lyz*	Lysozyme	F-TTCCAGATCAACAGCCGCTA R-GATCGCCACTGTGATGTCAT	111	1.94	81.87 ± 0.7	Yes
*Mvp*	Major vault protein	F-ACCAACTCCCAGGAGGCT R-CCTCTCCAGACGACCACG	75	1.97	78.93 ± 0.3	Yes
*Nattl*	Natterin-like protein	F-GTGAAAGTCACCTGCATGAATG R-CATCTCTCCTTTGTGGATACCC	104	1.98	78.81 ± 0.8	No
*Nattl-1*	Natterin-like protein paralog-1	F-AATCCGTGTCCTACCACAATGA R-GGTGTGTCGGTCAAAGCA	135	1.77	78.03 ± 0.1	No
*Nattl-2*	Natterin-like protein paralog-1	F-TGAAATVTVTGTCTCATCACAAC R-GGATCTGGTCGAGGTGGC	163	1.72	80.50 ± 0.2	No
*Nattl-3*	Natterin-like protein paralog-1	F-GTGACATCCGTTTCTCACCAG R-GATGTGTCGGTCAAAGCG	138	1.77	79.12 ± 0.2	No
*Ndub6*	NADH dehydrogenase 1 beta subcomplex subunit 6	F-TGGTGGAGTGTTCGCCTT R-CTCTCTGGGAGGTCTGGAA	171	1.89	82.40 ± 0.3	Yes
*Parp6*	Poly (ADP-Ribose) Polymerase Family, Member 6	F-CCGTATGAATACCGTTCCACAGG R-CACCCAGATGTTGCCGTGCTT	147	1.93	81.87 ± 0.7	Yes

**Supplemental Table S1 B.** 

Gene	Description	Primer Sequence (5’- 3’)	Product Size (bp)	PCR Efficiency	Melting Temperature (°C)	Exon Boundary
*Rarg*	Retinoic acid receptor gamma-A	F-AAGGCGAGCCCCTTCTTC R-TGCTCTGGGTCTCCACCG	82	1.92	78.62 ± 0.3	Yes
*Scin*	Scinderin/Adseverin	F-CACCTGATCCCAGACATCCAA R-CCTCACTCAACAACCTCGC	136	1.90	83.24 ± 0.7	No
*Tgfbr2*	TGF-beta receptor type-2	F-CTGCTCCGAGGACGAGTG R-ACCGACACCACCTGGGAG	72	1.93	79.02 ± 0.5	Yes
*Ubl5*	Ubiquitin-like protein 5	F-AATAAGGATGATTGAGGTGGTTTG R-ATGAGCTTCTTCAGGTCTCC	99	1.95	78.44 ± 0.3	Yes
*Ub2l3*	Ubiquitin-Conjugating Enzyme E2L 3	F-CGAGAAGGGACAGGTGTGTC R-ACCAACGCAATCAGGGACT	96	1.93	79.62 ± 0.3	Yes
*Vdra*	Vitamin D3 receptor A	F-CGTCACCAAGGCGGGTCA R-TGGAGCTTG AGTTTCTTCAGGC	81	1.93	78.12 ± 0.3	Yes

**Supplemental Table S2 A.** Verification of candidate polymorphisms. Primer sequences, melting temperatures and primary data.

Sequence	Position	Forward primer	Reverse primer	Tm forward	Tm reverse	Paralogs
NC_000861.1	1829	GTGCCTCAGACCCACCTAGA	TCTGTCGCCCGTACTAAGGT	60.26	59.76	No
NC_000861.1	3119	GGCCAGAGTAAACACCGAGA	CCTGGATTACTCCGGTCTGA	60.25	60.07	No
NC_000861.1	3411	GGCCAGAGTAAACACCGAGA	CCTGGATTACTCCGGTCTGA	60.25	60.07	No
NC_000861.1	8876	GACGTCCTTCACTCCTGAGC	GGGCTCATAAACTGGTCGAA	59.99	60.07	No
NC_000861.1	15240	ACCCTAAAACCGAACGATCC	TGGCTAGGAAGAGTCCGGTA	60.19	59.83	No
SS2U034121	233	CTCAACGTGCTTGACCAGTG	CCCTTACCCTCCAGGATCTC	60.5	59.89	Yes
SS2U054644	1037	AAGGACGGCCACTATGGTCT	GGGGCATAGAGTGCACAGG	60.9	61.65	Yes
SS2U054644	1188	TCAGAGATAGTGAAGAAGATGCTG	CGTACTTGATAAGACCTGTCGGTA	57.92	59.62	No
SS2U054644	1283	TCAGAGATAGTGAAGAAGATGCTG	CGTACTTGATAAGACCTGTCGGTA	57.92	59.62	No
SS2U055283	1822	TGTGTGAGGTGGTTGAGGAG	GGGTCATTGCTCCCTACAGA	59.7	60.07	No
SS2U055923	615	GTGGACCCAGAGGATGAGAA	AGAACCTGCTCCCAGTTTGA	60.05	59.84	No
SS2U058906	350	GCCAAAACCTCCACAATGAT	AACTGGCCTTCCAGATCAGA	59.8	59.8	Yes/No

Paralogs: indicates whether the PCR and sequencing yielded mixed products, indicative of paralogous genes.

**Supplemental Table S2 B.** 

Sequence	Genome contig	Gene name	Position	Ref	Var	Freq_AC	Freq_SB	FreqP_PL	FreqP_SB	FreqP_LB
NC_000861.1	n.a.	12S ribosomal RNA	1829	G	A	0 / 53	77 / 81	0 / 6	3 / 4	1 / 8
NC_000861.1	n.a.	16S ribosomal RNA	3119	A	T	46 / 87	18 / 28	0 / 8	0 / 8	0 / 8
NC_000861.1	n.a.	16S ribosomal RNA	3411	C	T	0 / 119	26 / 33	0 / 8	5 / 8	1 / 8
NC_000861.1	n.a.	tRNA-Lys	8876	C	A	73 / 779	74 / 352	0 / 4	0 / 4	n.a.
NC_000861.1	n.a.	NADH dehydrogenase 6	15240	G	A	2 / 3608	137 / 2702	2 / 4	0 / 4	n.a.
SS2U034121	AGKD01052493.1	Eukaryotic translation initiation factor 4 gamma 2	233	C	T	0 / 95	22 / 40	2 / 4	2 / 2	n.a.
SS2U054644	AGKD01031893.1	Uroporphyrinogen decarboxylase	1037	G	A	28 / 33	0 / 56	n.a.	n.a.	n.a.
SS2U054644	AGKD01031893.1	Uroporphyrinogen decarboxylase	1188	C	T	0 / 53	19 / 25	4 / 4	4 / 4	n.a.
SS2U054644	AGKD01031893.1	Uroporphyrinogen decarboxylase	1283	G	A	4 / 60	12 / 15	4 / 4	4 / 4	n.a.
SS2U055283	AGKD01013777.1	DNA2-like helicase	1822	G	A	1 / 65	25 / 50	3 / 4	n.a.	n.a.
SS2U055923	AGKD01022586.1	Bystin	615	G	A	106/109	7 / 190	0 / 4	n.a.	n.a.
SS2U058906	AGKD01005918.1	Mid1-interacting protein 1-like	350	G	T	0 / 49	67 / 68	4 / 4	4 / 4	n.a.

Sequence: name of the genebank sequence or EST-contig used as reference for mapped reads.

Genome contig: name of salmon genome (ICSASG_v1) contig with best sequence match to the respective EST-contig.

Ref: Reference variant.

Var: The derived variant.

Freq_AC and Freq_SB: Frequency of variant reads as fraction of total numbers of reads mapped in Aquaculture (AC) or Small benthic (SB).

FreqP: The frequency of variant in genotyping by PCR and direct sequencing, as a fraction of total number of chromosomes sequenced.

**Supplemental Table S3.** Mapping of Illumina reads to *S. salar* EST data. Numbers of reads aligning to salmon reference for each sample.

Alignment per read	SB 141	SB 163	SB 200	SB 433	AC 141	AC 163	AC 200	AC 433
0	33088778	30492314	27175901	25569628	32159386	30051365	31267710	28563169
1	6979368	11791558	11449549	11058555	11599602	11320997	11027195	10650748
2	2742358	4021683	3814418	3734404	4328402	4523686	3959198	3655786
3	2099068	2964994	2748108	2651522	3111277	3332577	2878729	2515303
4	1228292	1777846	1720902	1968251	1977738	2182392	1929818	1980420
5	914704	1317556	1284262	1434314	1471739	1679277	1447604	1426744
6	645264	946579	938290	1087959	1001350	1083025	1045157	1081063
7	425856	595785	578175	726290	657220	750523	690286	735351
8	293065	428003	424426	590100	530040	591332	527821	579860
9	206205	319401	334861	455838	296169	334264	387901	485653
10+	749074	1419362	1761275	3041930	1092980	1189781	1967857	3294222
Total reads	49372032	56075081	52230167	52318791	58225903	57039219	57129276	54968319

**Supplemental Table S4.** ANOVAs on qPCR data. Expression of nine genes was analyzed in whole SB- and AC-charr embryos, at two developmental timepoints (161 and 200 *τs*).

Gene	Term	Df	F value	p value	Significance	FDR RNA-seq
*Alp*	Morph	1	13.4797	0.0214	*	0.0697
	Time	1	14.9526	0.0180	*	0.0012
	M x T	1	3.9519	0.1177	.	
*Cgat2*	Morph	1	0.0257	0.8804	.	0.0035
	Time	1	1.5141	0.2859	.	0.3312
	M x T	1	0.1866	0.6880	.	
*Cox6B1*	Morph	1	0.0898	0.7793	.	0.0580
	Time	1	3.8312	0.1219	.	0.6320
	M x T	1	0.7359	0.4393	.	
*Krtap4–3*	Morph	1	30.0255	0.0054	**	0.0121
	Time	1	0.3902	0.5661	.	0.2784
	M x T	1	4.5225	0.1006	.	
*Lyz*	Morph	1	64.1566	0.0013	**	0.0406
	Time	1	1.0390	0.3657	.	0.0005
	M x T	1	1.2026	0.3344	.	
*Nattl*	Morph	1	8.1148	0.0465	*	7.718e-07
	Time	1	14.6659	0.0186	*	6.714e-14
	M x T	1	0.2958	0.6154	.	
*Ndub6*	Morph	1	0.7447	0.4368	.	0.0982
	Time	1	7.3316	0.0537	.	0.6698
	M x T	1	0.2269	0.6587	.	
*Parp6*	Morph	1	11.2682	0.0284	*	0.1076
	Time	1	0.7393	0.4384	.	0.3789
	M x T	1	0.2343	0.6537	.	
*Ubl5*	Morph	1	1.1420	0.3454	.	0.0587
	Time	1	0.2434	0.6476	.	0.0025
	M x T	1	0.3974	0.5627	.	

Significance: p > 0.05; * p < 0.05; ** p < 0.01.

FDR RNA-seq: indicates significance of Morph and Time effects in the transcriptome data.

**Supplemental Table S5.** ANOVAs on Natterin-like qPCR on adults. Studied were levels of *Natterin-like* and *Natterin-like Paralogs 1–3* in Arctic charr whole embryos (among SB, AC and PL morphs) and tissues from adult AC-charr.

Gene	Term	Df	F value	p value	Significance
*Nattl*	Morph	2	11.5515	0.0002	***
	Time	5	8.3202	3.99e-05	***
	M x T	9	4.4758	0.0007	***
*Nattl1*	Morph	2	19.4070	0.0001	***
	Time	3	5.9346	0.0089	**
	M x T	5	4.5761	0.0126	*
*Nattl2*	Morph	2	14.2921	0.0005	***
	Time	3	15.0463	0.0001	***
	M x T	5	3.2462	0.0404	*
*Nattl3*	Morph	2	34.4888	6.33e-06	***
	Time	3	4.4204	0.0238	*
	M x T	5	4.1843	0.0174	*
*Nattl*	Tissue	6	15.468	1.42e-08	***
*Nattl1*	Tissue	6	12.022	0.0002	***
*Nattl2*	Tissue	6	7.6811	0.0011	**
*Nattl3*	Tissue	6	46.182	8.89e-06	***

Significance: p > 0.05; * p < 0.05; ** p < 0.01.

**Supplemental Table S6.** Gene Ontology analyses of derived SNPs in SB-charr.

Category	Observed	In category	TERM	FDR adjusted p-value
GO:0006412	24	189	translation	4.34E-006
GO:0006396	8	32	RNA processing	0.0016
GO:0006414	6	19	translational elongation	0.0038
GO:0006313	5	20	transposition, DNA-mediated	0.0498
GO:0015074	5	21	DNA integration	0.0510
GO:0006260	6	35	DNA replication	0.0679
GO:0055114	20	285	oxidation-reduction process	0.0679

**Supplemental Table S7.** Predicted effect of SNP-candidates differing in frequency between charr morphs.

Effect on transcribed region	Uni_SB	Uni_AC	Rep_SB	Rep_AC
5´prime	32	19	35	24
Synonymous	232	179	176	113
Non-synonymous	112	72	81	72
3´prime	147	123	59	74

From RNA-reads that mapped to one (Uni) or more (Rep) *S. salar* ESTs.

The candidate SNPs frequencies differ more than 50% between SB and AC-charr, summarized by which morph with higher frequency of the derived allele.
