# Supplementary material for: The developmental transcriptome of contrasting Arctic charr ( Salvelinus alpinus) morphs
Source: F1000Res. 2016 Apr 25;4:136. Originally published 2015 Jun 1. [Version 2] doi: 10.12688/f1000research.6402.2 (PMC5007756; doi:10.12688/f1000research.6402.2)
Supplement: Peer review history

# Peer review: Referee response for version 2

**DOI:** 10.5256/f1000research.9044.r15587

**Published:** 2016-08-30

**Authors:** Örjan Östman

**Affiliations:** 1 Department of Aquatic Resources, Swedish University of Agricultural Sciences, Uppsala, Sweden

The study by Gudbrandsson et al. reports a thorough analysis of differences in the transcriptome between different ‘morphs’ or ‘populations’ of artic charr. More specifically they have studied the transcriptome of eggs and larvae from a natural population of small benthic charr (SB) and Icelandic aquaculture charr (AC), which is fast growing and have a ‘limnic-like’ morphology. They find a list of potential candidate genes involved in the ecological differentiation of artic charr (and during the embryonic development). In addition they studied the transcriptome from different tissues of adult AC-charr. From the transcriptome of these populations two populations they developed 12 SNP-markers applied to other sympathric (Lake Thingvallavatn) wild morphs to study if these genes differed between other morphs. Finally they also study mtDNA expression between morphs to find they mainly differ between the benthic morphs and a limnic.

 The search for genes involved in the ecological divergence of species is an important topic that has exploded the last decade with the new generation of sequencing. I find this study to be an important contribution because of the study system with artic charr is an example of relative recent and rapid divergence into many different morphs/ecological, and the extensive and thorough investigation of the differences in the transcriptome between morphs.

 However, I think the authors try to stretch their conclusions a bit too far. The study is great as a base for further research in the topic, which I guess is in the pipeline. The use of cultivated charr make sense for comparing the most extreme morphs. But to me it does not make sense for making conclusions about genes involved in the ecological niche differentiation in natural populations, which is the motivation of the study in the introduction and brought up in the discussion). The cultivated population has been selected for fast body growth and they are not from Lake Thingvallavatn and little can therefore be said about the genetics of the ecological differentiation of sympatric species. Not very surprising genes related to metabolism seemed upregulated in AC and immunogens upregulated in SB. What does that actually tells us about the genetics of ecological differentiation of natural populations??

 Although the aims on p. 4 feels valid, they are not contingent with the previous text in the introduction. Thus, I suggest that the much of the earlier part of the introduction is rewritten to actually address the differences in gene expression between a cultivated morph and its extreme opposite small benthic artic charr.

 As far as I understand it is only egg that are kept in the same environment, but the parents have been raised in different environments and transgenerational plasticity cannot be ruled out. This is not a major criticism (the ideal case would be to have had them in lines in a common environment of course) but needs to be addressed in the text.

 The ‘Nattl’ paralogs provide an interesting case where the expression of different paralogs has been studied. But again, are the result difficult to interpret from an ecological niche differentiation perspective. Often is the natural small limnic morph (PL) in between SB and AC (Fig. 5A), but for Nattl1 and Nattl2 AC and SB seem most similar? The connection to the original question is weak and the authors do not conclude more than “…it is not divergence in the expression of one paralog that explains the general nattl expression disparity in the transcriptome.” Fair enough, but that is more about the genetic architecture than the genetics of ecological divergence.

 In the validation of the transcriptome differences with qPCR of 9 genes/paralogs only 5 was still significant. What conclusions should one make out of that, that around half of the 296 paralogs differing between SB and AC are false detection (despite FDR < 5%). I support the use of qPCR but please comment on the implication of this.

 I think Fig. 6 should be converted into a bar-graph plot instead (this feels more like a table).

 To conclude, I think this study is a great contribution for suggesting putative differences in the transcriptome of a fish species. However, the importance for understanding ecological differentiation of sympatric species it is, however, so far limited as it that would require more using natural morphs, replicated populations, back-crosses, investigation of plasticity and how reproductive isolation is maintained etc., which is likely to come. But until that, I suggest the this text should mainly focus on the differences between SB and AC arctic charr, and not try to squeeze in everything in one paper.

 Minor comments:

 In the equation on p. 6 I guess M & T is ‘Morph’ and ‘Time’? If so spell out or use M & T consistently.

 Note that on p. 8 the qPCR of 8 paralogs in embryonic heads is metioned but the results do not come until “Expression differences in the developing heads of benthic and limnetic charr morphs”.

 Use “.” instead of “,” as decimal sign in Fig. 4.

I have read this submission. I believe that I have an appropriate level of expertise to confirm that it is of an acceptable scientific standard, however I have significant reservations, as outlined above.

# Peer review: Referee response for version 2

**DOI:** 10.5256/f1000research.9044.r13702

**Published:** 2016-05-25

**Authors:** Daniel Macqueen

**Affiliations:** 1 Institute of Biological and Environmental Sciences, University of Aberdeen, Aberdeen, UK

Second review of Gudbrandsson *et al*. “ *The developmental transcriptome of contrasting Arctic charr (Salvelinus alpinus) morphs”.*

**Overview:**

 The authors have addressed the comments made by myself and Anne Dalziel. They have incorporated a range of associated changes into version 2 of their paper. Readers will find these changes, along with several clarifications provided in the published response to reviewers section, to facilitate transparent interpretation of this large and diverse study, including its strengths and caveats. My overall opinion of the study remains unchanged – it is interesting and reports findings of merit that will be followed up on in future work. **I am thus happy to approve version 2 of the paper**.

 I did spot a few typos or grammatical issues that the authors might address and had some final comments that might be addressed – all of a minor nature and easy to address.

- Abstract – “ *energy metabolism and blood coagulation genes*” remove “ *genes*”
- Abstract -  “ *Comparison of single nucleotide polymorphism (SNP) frequencies reveals*” change “ *reveals*” to “ *revealed*” (for accurate use of tense)
- Introduction – “ *Examples of such species complexes are provided finches of the Galapagos island”* should be “ *Examples of such species complexes are provided by finches of the Galapagos island*”
- Introduction: “ *Thus we were quite keen to apply RNA-sequencing to analyze ecomorphs in our study system, Arctic charr*”. The authors should add the Latin name for charr here, rather than in the next paragraph.
- Introduction: “ *The family is estimated to be between 88–103 million years old ^21,22^. A whole genome duplication event occurred before the radiation of the salmonid family ^21–24 ^which has provided time for divergence of ohnologous genes (paralogous genes originated by whole genome duplication event*”

 

 It would be simpler to just state that the common ancestor to salmonids experienced a whole genome duplication 88–103 million years ago. The actual age of the salmonid family depends on whether one considers the (extinct) direct ancestors to salmonids that didn’t experience genome duplication to be salmonids.

  

- Introduction: “ *Furthermore, recent estimates from the rainbow trout (Oncorhynchus mykiss) genome suggest that ohnologous genes are lost at a rate of about 170 genes per million years, and that on the order of 4500 were retained in rainbow trout ^22^*”

 

 This information is inaccurate. Firstly, based on the paper cited (Berthalot *et al*. 2014), this information should state that around 4,500 *pairs* of ohnologous genes were retained from Ss4R (i.e. around 9,000 separate genes). More importantly, without going into detail, the stated data represents a non-comprehensive fraction of the genome. I suggest the authors update this part of the text with accurate estimates, since the number of retained Ss4R ohnologue pairs in much larger than what is stated. The authors might also draw in more comprehensive data from the recent publication of the Atlantic salmon genome (Lien et al. Nature, 533, 200–205) ^1^. The simplest way to present the information is to state that around half of the original Ss4R ohnologue pairs are still functionally retained (both stated papers are in agreement about that).

  

- Figure 1: It would be easier for the reader to link the text and images if the authors updated with ‘a’, ‘b’, ‘c’ and ‘d’ panels for each of the different charr morphs.
- Introduction: “ *In this study, we compare SB-charr from*” should be “ *In this study, we compared SB-charr from*” (again, it is correct here to use past tense – the authors should check the rest of the manuscript for similar tense issues).
- Figure 2: Minor comments – the text “ *Map on salmon genes*” is vague and open to several interpretations. Better: “ *Map on Atlantic salmon expressed sequence tags*”?

I have read this submission. I believe that I have an appropriate level of expertise to confirm that it is of an acceptable scientific standard.

## References

[1] LienSKoopBFSandveSRMillerJRKentMPNomeTHvidstenTRLeongJSMinkleyDRZiminAGrammesFGroveHGjuvslandAWalenzBHermansenRAvon SchalburgKRondeauEBDi GenovaASamyJKOlav VikJVigelandMDCalerLGrimholtUJentoftSInge VågeDde JongPMoenTBaranskiMPaltiYSmithDRYorkeJANederbragtAJTooming-KlunderudAJakobsenKSJiangXFanDHuYLiberlesDAVidalRIturraPJonesSJJonassenIMaassAOmholtSWDavidsonWS: The Atlantic salmon genome provides insights into rediploidization. *Nature*.2016;533(7602) : 10.1038/nature17164 200-5 10.1038/nature17164 27088604PMC8127823

# Peer review: Referee response for version 1

**DOI:** 10.5256/f1000research.6869.r9419

**Published:** 2015-07-09

**Authors:** Anne Dalziel

**Affiliations:** 1 Institute for Systems and Integrative Biology (IBIS), Department of Biology, Laval University, Quebec City, QC, Canada

In this paper “The developmental transcriptome of contrasting Arctic charr ( *Salvelinus alpinus*) morphs” Gudbrandsson *et al*. have tested for differential gene expression at multiple developmental time-points among a number of Artic charr morpho-types from Lake Thingvallavatn (3 wild morphs, 1 studied with RNA-seq and qPCR, the others with qPCR only) and Holar aquaculture (1 domesticated morph, RNA-seq and qPCR).  They have also studied multiple tissues/body regions for a subset of the differentially expressed genes found with RNA-seq.  The goal of the paper was to find candidate genes that may underlie variation in morphology, with a focus on craniofacial morphology related to benthic vs. limnetic feeding. In general, I think this goal was met and this paper contributes to our understanding of the mechanisms contributing to morphological evolution in a non-genetic model organism. The authors provide an extensive, multi-time point comparison of two morphologically divergent groups of charr reared in a common environment (reducing the influence of phenotypic plasticity) and have collected a tremendous amount of data. This information will help them to hone in on the genetic loci contributing to phenotypic evolution in this very interesting system, and on the effects of domestication. However, there are a number of major issues that do need to be more clearly addressed in the manuscript prior to final publication. I have outlined these comments below.

**Major Comments**

1. Introduction:
  Requires some reorganization, clarification of what phenotypes have evolved in parallel among morphs, and how the authors separate the effects of domestication (SB vs. AC) from benthic/limnetic evolution (SB/LB vs. PL/AC).
  a) At present, the introduction focuses upon the utility of instances of parallel evolution to help us determine how repeatable evolutionary change may be. This is definitely true, and the repeated evolution of the dwarf, benthic morph (SB; the focus of the introduction/abstract/discussion) in many lakes strongly argues that this phenotype has evolved via natural selection. However, it is not clear to me if true ‘parallelism’ seen among the SB (small benthic) and LB (large benthivorous) vs. AC (Holar aquaculture) and PL (small planktivorous) morphs because not enough information is provided for me to assess this. To support the argument for parallelism the specific traits that have evolved in parallel among morphs must be displayed and the evolutionary history of these morphs should be clarified (e.g. in paragraph 6 and Figure 1). As well, any related non-parallelism in traits should also be discussed (i.e. how are the domesticated AC and wild PL different?). At present Figure 1 only shows the AC and SB morphs, and does not point out the specific traits they are interested in. This is critical background information for readers who are not familiar with this system.
  b) The comparison of AC (domestic, limnetic-like head) vs. LB (wild, benthic like head) looks at two confounded variables: domestication and the benthic/limnetic morphology. This should be clearly stated in the introduction, and the use of the additional morphs (PL, LB) in detangling domestication vs. benthic/limnetic evolution should be noted.
  c) The use of the AC morph is still a bit unclear to me. The argument for point ‘ii) of the availability of abundant AC material’ could be expanded by providing more information on the ‘limnetic’ like features of this morph and why it is an appropriate comparison to a benthic morph, the genetic divergence from the lake Thingvallavatn fish, and also the selection regime it has experienced (selection for limnetic features? What other traits vary with domestication?).
  d) Paragraph 2 – Much of this paragraph, including discussing the ability to measure gene expression and relate to phenotype in fishes, is unnecessary as fish are no different from other vertebrates in this respect. Instead, the final sentence “One approach to identify pathways related to function or morphological differences is to study gene expression during development” should become the ‘topic sentence’ and expanded upon to explain why gene expression studies are especially relevant ways to link genotype to phenotype in evo-devo studies.
  e) Better highlight the strengths – The authors have done a wonderful job of assessing multiple developmental time points and rearing fish in a common garden environment. However, they do not highlight these strengths. Some small notes on the importance of controlling for phenotypic plasticity in these traits (which are known to be quite plastic) to better study genetic differentiation would be a nice addition.
2. Methods:
  a) Page 4 paragraph 1 - Clarify the number of fish used to make the crosses (this will help us determine the likelihood of selecting a full or half-sib for sequencing/qPCR).
  b) I should note that I am not an expert in the analysis of RNA-seq data, but luckily the first reviewer has done an excellent job of commenting upon these aspects of the project. I fully agree with their comments and suggestions. I would also like to see more information on the methods used to pool samples and how RNA-seq data was normalized among samples, developmental times and morphs. I will also note that the authors often use *S.salar* for comparisions, not *O.mykiss*, which is a closer relative to *S.alpinus*. The reasons for this approach should be discussed.
  c)  I am also not trained as a population geneticist. However, from my experience studying paralogous genes in salmonids, and with respect to the author’s own findings for the Nattl paralogs (Fig 4), I do not think it is prudent to “assume that the expression of paralogous genes is stable… ” in the methods (page 12).  In fact, Berthelot *et al*. (2014) find the opposite (see my comments for the discussion).
  d) The authors should use their genetic information to test if the fish chosen are siblings with each other (full or half-sibs). This may have important implications for the population genetic analyses.
  e) Page 5 - It is not appropriate to change the meaning of the word ‘gene’. I think it is much clearer to use the term ‘paralog group’ or ‘gene family’ when referring to the fact that the authors do not study single genes, but instead groups of paralogs.
  f) Selection of genes for qPCR – the methods by which genes for the qPCR studies (Fig 3) were selected should be clearly noted. From my reading, it seems that most of these genes do not significantly vary among SB and AC at the 1% FDR level (Tables 1 and 2; only Natterin?). Thus, I am assuming these genes are only significant at the 5% FDR level (S1 file) – why focus upon these and not those significant at 1%?  As well, it would be good to include information on why different genes were selected for Figure 3 (qPCR validation of whole fish) and Figure 4 (candidate genes-qPCR validation in just the head). Finally, the abbreviations used for qPCR validation should also be listed in Table 1 for easy comparisons among figures/tables.
3. Results & Figures:
  a) Include an experimental design figure - At present, it is difficult to keep track of all of the morphotypes, tissues, and developmental time points used without referring to the methods. Thus, an experimental design figure summarizing the samples used (morphotype, population, sample size, developmental time point), how they were pooled and which techniques were used to measure gene expression on each sample (RNA-seq and/or qPCR)  is needed.
  b) Include the LB and PL morphs in Figure 1 and clarify traits of interest – The legend states that “differences in size, coloration and head morphology are apparent”, but it would be better to specifically point out the differences they are referring to. F1000 is for a general audience, and this would help non-ichthylogists better understand what ecologically-important traits the authors are interested in (e.g. those related to benthic/limnetic feeding).  In addition, the two other morphs used in the qPCR studies should also be displayed (large benthivorous and small planktivorous) to facilitate phenotypic comparisons and assess parallelism in benthic/limnetic feeding and/or the effects of domestication on AC.
  c) Figure 5- this is actually a table not a figure (?) and is a bit confusing. I think it is much easier to interpret Figure S2 (displaying the data as in Fig 3 and 4), and that Fig 5 and S2 should be switched. It would be great to show significant differences in mRNA content in this, and all other figures, by including symbols. Also, full gene names should be listed in all figure legends.
4. Discussion:
  The discussion focuses on the SB morph (page 17 – “The objective of this study were to get a handle on genetic and molecular systems that associate with benthic morphology in charr by mainly focusing on the small benthic morph in Lake Thingvallavatn, Iceland”), while the introduction discusses parallel evolution (indicating that the comparisons should be among many morphs). These are two different topics i) mRNA content differences among benthic vs. limnetic morphs changing in parallel or ii) linking mRNA content to phenotype in SB (benthic, wild) vs. AC (limnetic head, domesticated) morphs. In particular, the role of domestication vs. wild fish divergence needs to be addressed. At present these two topics/questions are mixed in the introduction/discussion and should be addressed separately.
  a) Paragraph on Immune Defences - Is immunity also expected to evolve in parallel in all benthic morphs? Is this predicted to be unique to SB vs. AC? Whatever the case, the parallelism (or not) in these genes should also be discussed, and whether this relates more to domestication in AC or differences between limnetic vs. benthic fish.  Much of the functional discussion can also be cut.
  b) Page 18 – The information about genes found to be differentially expressed among morphs in your prior work should also be in the introduction, as it is background work that explains why you took this transcriptomic approach. This can also be used to explain why you focused in on particular qPCR genes.
  c) A discussion of domestication related differences vs. benthic/limnetic differences should be included. I think the data from head gene expression is very interesting (Figs 5, S2) and really speaks to this question.
  d) In general, the role of stochastic evolutionary processes, and not just selection (artificial and natural) should be noted. For example, if the AC charr were simply taken from a stock with a different mtDNA haplotype then these differences in the mtDNA genome might not be adaptive, just random.  If the AC fish has much higher mtDNA expression might this be simply a domestication issue and not indicative of selection in SB as stated?   Finally, you find that not all mitochondrial transcripts (which are transcribed as a polycistronic transcript) are found at similar levels (Table 1) – what does this tell you about differential degradation/post-transcriptional processes?
  e) There is no discussion about the “Analyses of polymorphism in Arctic charr transcriptome” (Table 3, 4, 5), except for the mtDNA.

 

**Minor Comments**

Introduction:

a) Paragraph 3 – “Furthermore, recent estimates from the rainbow trout….by utilizing multiple data sources the genome assembly problem of this family can be solved”. I am not sure how this statement is relevant to this particular study. This and the following statement seem more appropriate for the methods/discussion to me.

b) The morphs being discussed should be clarified throughout the paper. For example, the authors often state “among morphs/among charr populations” but it is not clear which of the many morphs they are referring to (e.g. Paragraph 5, first sentence on allozymes and mtDNA and later sentence on MCHIIa – do you mean all 4 morphs of specific 2-way comparisons? Are some morphs more differentiated than others?)

Methods:

a) The authors should note why they did not use the PI (large piscivorous) morph in any qPCR studies (in the methods or discussion) as this would be a nice morph to use in their tests for parallelism.

b) Page 5 (last paragraph) – the methods used to remove particular variants needs to be clarified. In particular, why the assumptions used to remove variants are valid by referencing past studies.

Figures & Results:

a) Figure 2. The key for Figure 2 should include a specific heading for morph and time-point with the abbreviations restated [e.g. Timepoint: 141 dpf, Morph: Small Benthic (SB)].

b) Figure 6 – would be helpful to label the protein coding genes in this figure as well as the 12s and 16s RNAs.

c) Figure 7 – It is not clear to me which variant is present in which morph. Adding the nucleotide to the x-axis (i.e. frequency of m1829G for B) would make this figure easier to quickly interpret. The “A.charr_WT” and “A.charr_M” should also be defined in the legend and it would be more appropriate to use scientific names for all species.

Discussion:

a) Discussion of reference 32 – The discussion of reference 32 is not put into the proper context. Figure 6 of this paper (Berthelot *et al*. 2014) shows that there are many genes that have no correlation among expression patterns and/or differences in expression levels (1573, 1248, and 1895=4716 paralog pairs), and that together these represent more than the 1,407 correlated/similar expression level paralogs. This section of the discussion needs to be modified.

b) The Norman *et al*. (2014) paper should be mentioned earlier – if this is available why was it not used for their analyses? As well, the last sentence in this paragraph can be cut as it is evident.

c) Page 18 – “Our new data also demonstrate differences in craniofacial elements between AC- and SB-charr, along a limnetic vs. benthic axis ^79^”. Are you referring to ref 79 or data from this study? If you are referring to 79, clarify and note what you found. This occurs a few times in the discussion

General grammatical errors

There are a number of grammatical errors throughout this paper (e.g. “31 genes were higher expressed in SB and 40 genes higher in AC-charr”; “that may help sculpture benthic vs. limnetic heads” pg 19).

I have read this submission. I believe that I have an appropriate level of expertise to confirm that it is of an acceptable scientific standard, however I have significant reservations, as outlined above.

# Response

**Published:** 2016-04-04

**Authors:** Arnar Palsson

**Affiliations:** University of Iceland, Iceland

*Major Comments*

*Introduction:‭*

*‬Requires some reorganization,‭ ‬clarification of what phenotypes have evolved in parallel among morphs,‭ ‬and how the authors separate the effects of domestication‭ (‬SB vs.‭ ‬AC‭) ‬from benthic/limnetic evolution‭ (‬SB/LB vs.‭ ‬PL/AC‭)‬.‭*

*‬a‭) ‬At present,‭ ‬the introduction focuses upon the utility of instances of parallel evolution to help us determine how repeatable evolutionary change may be.‭ ‬This is definitely true,‭ ‬and the repeated evolution of the dwarf,‭ ‬benthic morph‭ (‬SB‭; ‬the focus of the introduction/abstract/discussion‭) ‬in many lakes strongly argues that this phenotype has evolved via natural selection.‭ ‬However,‭ ‬it is not clear to me if true‭ ‘‬parallelism‭’ ‬seen among the SB‭ (‬small benthic‭) ‬and LB‭ (‬large benthivorous‭) ‬vs.‭ ‬AC‭ (‬Holar aquaculture‭) ‬and PL‭ (‬small planktivorous‭) ‬morphs because not enough information is provided for me to assess this.‭ ‬To support the argument for parallelism the specific traits that have evolved in parallel among morphs must be displayed and the evolutionary history of these morphs should be clarified‭ (‬e.g.‭ ‬in paragraph‭ ‬6‭ ‬and Figure‭ ‬1‭)‬.‭ ‬As well,‭ ‬any related non-parallelism in traits should also be discussed‭ (‬i.e.‭ ‬how are the domesticated AC and wild PL different‭?)‬.‭ ‬At present Figure‭ ‬1‭ ‬only shows the AC and SB morphs,‭ ‬and does not point out the specific traits they are interested in.‭ ‬This is critical background information for readers who are not familiar with this system.*

**Reply:**‭ ‬These are excellent suggestions.‭ ‬At the end of the intro we stress the difference between the aims of our research program‭ (‬study the genetics of parallel evolution‭) ‬and the aims of this study‭ (‬get a handle on differences between sympatric morphs,‭ ‬with the AC as possible outgroup‭)‬.‭ ‬The morphs studied here do not represent parallel evolution of benthic phenotypes‭ (‬SB and LB are both from the same lake and appear to be closely related‭ ‬-‭ ‬Kapralova et al‭ ‬2011‭)‬.‭ ‬Analyses of that question requires further studies.‭ ‬This data can implicate genes that separate PL/AC and SB/LB and may be studied in such follow up analyses of more populations.‭ ‬We have updated figure‭ ‬1‭ ‬as advised‭ ‬-‭ ‬including the‭ ‬4‭ ‬morphs studied,‭ ‬expanded on the legend and also provide an overview of research approach‭ (‬part B‭)‬.‭

‬ *b‭) ‬The comparison of AC‭ (‬domestic,‭ ‬limnetic-like head‭) ‬vs.‭ ‬LB‭ (‬wild,‭ ‬benthic like head‭) ‬looks at two confounded variables:‭ ‬domestication and the benthic/limnetic morphology.‭ ‬This should be clearly stated in the introduction,‭ ‬and the use of the additional morphs‭ (‬PL,‭ ‬LB‭) ‬in detangling domestication vs.‭ ‬benthic/limnetic evolution should be noted.‭*

*‬c‭) ‬The use of the AC morph is still a bit unclear to me.‭ ‬The argument for point‭ ‘‬ii‭) ‬of the availability of abundant AC material‭’ ‬could be expanded by providing more information on the‭ ‘‬limnetic‭’ ‬like features of this morph and why it is an appropriate comparison to a benthic morph,‭ ‬the genetic divergence from the lake Thingvallavatn fish,‭ ‬and also the selection regime it has experienced‭ (‬selection for limnetic features‭? ‬What other traits vary with domestication‭?)‬.‭*

**Reply** (‬b and c‭)‬:‭ ‬The reviewer is correct,‭ ‬AC and SB are separated by multiple traits,‭ ‬and the data probably reveal signals associating with most of them.‭ ‬Unfortunately the AC charr is not well characterized phenotypically,‭ ‬thus we can not address the question of other traits.‭ ‬We focus mainly on the head and jaw morphology,‭ ‬as these attributes distinguish benthic and limnetic morphs.‭ T‬he revised intro elaborates on the choice of AC,‭ ‬and how the follow up work on the morphs from Lake Thingvallavatn can help us sort this out.‭ ‬This point is also picked up in the discussion.

*d‭) ‬Paragraph‭ ‬2‭ – ‬Much of this paragraph,‭ ‬including discussing the ability to measure gene expression and relate to phenotype in fishes,‭ ‬is unnecessary as fish are no different from other vertebrates in this respect.‭ ‬Instead,‭ ‬the final sentence‭ “‬One approach to identify pathways related to function or morphological differences is to study gene expression during development‭” ‬should become the‭ ‘‬topic sentence‭’ ‬and expanded upon to explain why gene expression studies are especially relevant ways to link genotype to phenotype in evo-devo studies.‭*

**Reply**: ‬We restructured and shortened this paragraph around this topic sentence‭ ‬-‭ ‬and gave more room for the previous RNAseq study on Arctic charr.

‬ *e‭) ‬Better highlight the strengths‭ – ‬The authors have done a wonderful job of assessing multiple developmental time points and rearing fish in a common garden environment.‭ ‬However,‭ ‬they do not highlight these strengths.‭ ‬Some small notes on the importance of controlling for phenotypic plasticity in these traits‭ (‬which are known to be quite plastic‭) ‬to better study genetic differentiation would be a nice addition.*

**Reply**: ‬Great advice,‭ ‬we tried to integrate this into the last paragraph of the intro.‭

‬

*Methods:‭*

*‬a‭) ‬Page‭ ‬4‭ ‬paragraph‭ ‬1‭ ‬-‭ ‬Clarify the number of fish used to make the crosses‭ (‬this will help us determine the likelihood of selecting a full or half-sib for sequencing/qPCR‭)‬.*

**Reply**: ‬We did bulk crosses,‭ ‬joining eggs from‭ ‬5-10‭ ‬females in a can and sperm from‭ ‬3-5‭ ‬males‭ (‬SB,‭ ‬PL,‭ ‬LB‭) ‬and single parent cross for AC.‭ ‬Each sample included RNA pooled from‭ ‬3‭ ‬embryos,‭ ‬so there is a chance that full sibs were sequenced,‭ ‬but unlikely.‭ ‬The embryos/samples for qPCR are from similar pools. Now described better in methods.‭

‬ *b‭) ‬I should note that I am not an expert in the analysis of RNA-seq data,‭ ‬but luckily the first reviewer has done an excellent job of commenting upon these aspects of the project.‭ ‬I fully agree with their comments and suggestions.‭ ‬I would also like to see more information on the methods used to pool samples and how RNA-seq data was normalized among samples,‭ ‬developmental times and morphs.‭ ‬I will also note that the authors often use‭ ‬S.salar for comparisions,‭ ‬not‭ ‬O.mykiss,‭ ‬which is a closer relative to S.alpinus.‭ ‬The reasons for this approach should be discussed.‭*

**Reply**: ‬The RNA was isolated from individual embryos,‭ ‬quantified and then united‭ (‬in equal concentrations‭) ‬prior to cDNA synthesis.‭ The read counts per gene are normalized per million reads in sample. Not normalized with other variables.

‬ *c‭) ‬ I am also not trained as a population geneticist.‭ ‬However,‭ ‬from my experience studying paralogous genes in salmonids,‭ ‬and with respect to the author’s own findings for the Nattl paralogs‭ (‬Fig‭ ‬4‭)‬,‭ ‬I do not think it is prudent to‭ “‬assume that the expression of paralogous genes is stable‭… ” ‬in the methods‭ (‬page‭ ‬12‭)‬. ‭ ‬In fact,‭ ‬Berthelot‭ ‬et al.‭ (‬2014‭) ‬find the opposite‭ (‬see my comments for the discussion‭)‬.‭*

‬ **Reply**:‭ ‬Excellent suggestion.‭ ‬We corrected our misunderstanding,‭ ‬added this fact into the intro and discussion,‭ ‬and reinterpreted our data in this light.

*d‭) ‬The authors should use their genetic information to test if the fish chosen are siblings with each other‭ (‬full or half-sibs‭)‬.‭ ‬This may have important implications for the population genetic analyses.‭*

**Reply**:‬The fish chosen for pop-gen work are random sample from spawning grounds‭ ‬-‭ ‬assumed to be not sibling groups.‭ ‬Our earlier study‭ (K‬apralova‭ ‬2011‭) ‬showed no family structure in charr collected this way from the lake.

*‬e‭) ‬Page‭ ‬5‭ ‬-‭ ‬It is not appropriate to change the meaning of the word‭ ‘‬gene‭’‬.‭ ‬I think it is much clearer to use the term‭ ‘‬paralog group‭’ ‬or‭ ‘‬gene family‭’ ‬when referring to the fact that the authors do not study single genes,‭ ‬but instead groups of paralogs.*

‬ **Reply**: ‬Excellent suggestion.‭ ‬We amended this., and use paralog group throughout.

*f‭) ‬Selection of genes for qPCR‭ – ‬the methods by which genes for the qPCR studies‭ (‬Fig‭ ‬3‭) ‬were selected should be clearly noted.‭ ‬From my reading,‭ ‬it seems that most of these genes do not significantly vary among SB and AC at the‭ ‬1%‭ ‬FDR level‭ (‬Tables‭ ‬1‭ ‬and‭ ‬2‭; ‬only Natterin‭?)‬.‭ ‬Thus,‭ ‬I am assuming these genes are only significant at the‭ ‬5%‭ ‬FDR level‭ (‬S1‭ ‬file‭) – ‬why focus upon these and not those significant at‭ ‬1%‭?‬ ‭ ‬As well,‭ ‬it would be good to include information on why different genes were selected for Figure‭ ‬3‭ (‬qPCR validation of whole fish‭) ‬and Figure‭ ‬4‭ (‬candidate genes-qPCR validation in just the head‭)‬.‭ ‬Finally,‭ ‬the abbreviations used for qPCR validation should also be listed in Table‭ ‬1‭ ‬for easy comparisons among figures/tables.‭*

**Reply**: ‬Very important point.‭ ‬We deliberately studied some genes with less statistical support‭ (‬FDR between‭ ‬5%‭ ‬and‭ ‬10%‭)‬,‭ ‬to gauge the differences in the genes with less support and in particular to have a bigger pool of candidates that may relate to the specific developmental process‭ (‬like head and jaw formation‭)‬.‭ ‬Of course we can not assert that all the genes with strongest DE signal in the transcriptome are true positives,‭ ‬but the data can be used for hypothesis generation.‭ ‬We also amended table‭ ‬1‭ ‬and the figure legends accordingly.

*Results‭ & ‬Figures:‭ ‬*

*a‭) ‬Include an experimental design figure‭ ‬-‭ ‬At present,‭ ‬it is difficult to keep track of all of the morphotypes,‭ ‬tissues,‭ ‬and developmental time points used without referring to the methods.‭ ‬Thus,‭ ‬an experimental design figure summarizing the samples used‭ (‬morphotype,‭ ‬population,‭ ‬sample size,‭ ‬developmental time point‭)‬,‭ ‬how they were pooled and which techniques were used to measure gene expression on each sample‭ (‬RNA-seq and/or qPCR‭)‬ ‭ ‬is needed.*

*b‭) ‬Include the LB and PL morphs in Figure‭ ‬1‭ ‬and clarify traits of interest‭ – ‬The legend states that‭ “‬differences in size,‭ ‬coloration and head morphology are apparent‭”‬,‭ ‬but it would be better to specifically point out the differences they are referring to.‭ ‬F1000‭ ‬is for a general audience,‭ ‬and this would help non-ichthylogists better understand what ecologically-important traits the authors are interested in‭ (‬e.g.‭ ‬those related to benthic/limnetic feeding‭)‬. ‭ ‬In addition,‭ ‬the two other morphs used in the qPCR studies should also be displayed‭ (‬large benthivorous and small planktivorous‭) ‬to facilitate phenotypic comparisons and assess parallelism in benthic/limnetic feeding and/or the effects of domestication on AC.*

**Reply**: (‬a and b‭) ‬Excellent suggestions.‭ ‬Now picture‭ ‬1‭ ‬has all‭ ‬4‭ ‬morphs,‭ ‬and a schematic describing the work flow and samples.‭

*‬c‭) ‬Figure‭ ‬5-‭ ‬this is actually a table not a figure‭ (?) ‬and is a bit confusing.‭ ‬I think it is much easier to interpret Figure S2‭ (‬displaying the data as in Fig‭ ‬3‭ ‬and‭ ‬4‭)‬,‭ ‬and that Fig‭ ‬5‭ ‬and S2‭ ‬should be switched.‭ ‬It would be great to show significant differences in mRNA content in this,‭ ‬and all other figures,‭ ‬by including symbols.‭ ‬Also,‭ ‬full gene names should be listed in all figure legends.‭*

**Reply**: ‬We acknowledge that this graph is not the simplest,‭ ‬but would like to keep it over Figure S2.‭ ‬Our reasoning is that this graph illustrates the sharp differences between the limnetic‭ (‬AC-PL‭) ‬and benthic‭ (‬SB-LB‭)‬,‭ ‬which are the main result in this section.‭ ‬But we will of course switch them,‭ ‬or possibly join both in a single figure ‭?? ‬if the reviewer insists or the editors recommend it.

*Discussion:‭*

*‬The discussion focuses on the SB morph‭ (‬page‭ ‬17‭ – “‬The objective of this study were to get a handle on genetic and molecular systems that associate with benthic morphology in charr by mainly focusing on the small benthic morph in Lake Thingvallavatn,‭ ‬Iceland‭”)‬,‭ ‬while the introduction discusses parallel evolution‭ (‬indicating that the comparisons should be among many morphs‭)‬.‭ ‬These are two different topics i‭) ‬mRNA content differences among benthic vs.‭ ‬limnetic morphs changing in parallel or ii‭) ‬linking mRNA content to phenotype in SB‭ (‬benthic,‭ ‬wild‭) ‬vs.‭ ‬AC‭ (‬limnetic head,‭ ‬domesticated‭) ‬morphs.‭ ‬In particular,‭ ‬the role of domestication vs.‭ ‬wild fish divergence needs to be addressed.‭ ‬At present these two topics/questions are mixed in the introduction/discussion and should be addressed separately.‭*

**Reply**: ‬We tried to separate these two aims more clearly in the revised discussion.‭ ‬The strategy was to use the AC vs SB contrast for hypothesis generation,‭ ‬as the first aim is central to our program.‭ ‬We have now added sentences on the domestication in two parts of the discussion.

*‬a‭) ‬Paragraph on Immune Defenses‭ ‬-‭ ‬Is immunity also expected to evolve in parallel in all benthic morphs‭? ‬Is this predicted to be unique to SB vs.‭ ‬AC‭? ‬Whatever the case,‭ ‬the parallelism‭ (‬or not‭) ‬in these genes should also be discussed,‭ ‬and whether this relates more to domestication in AC or differences between limnetic vs.‭ ‬benthic fish. ‭ ‬Much of the functional discussion can also be cut.*

**Reply**: ‬Good question,‭ ‬we assume it to be so,‭ ‬but that may be wrong.‭ ‬We moved the discussion towards this question and away from functional description.‭

*‬b‭) ‬Page‭ ‬18‭ – ‬The information about genes found to be differentially expressed among morphs in your prior work should also be in the introduction,‭ ‬as it is background work that explains why you took this transcriptomic approach.‭ ‬This can also be used to explain why you focused in on particular qPCR genes.*

**Reply:** ‬We added a sentence in the intro about the published papers,‭ ‬that this transcriptome made available.‭ ‬In those papers we focused on genes with putative craniofacial effects,‭ ‬though the focus in this study was broader.‭

‬ *c‭) ‬A discussion of domestication related differences vs.‭ ‬benthic/limnetic differences should be included.‭ ‬I think the data from head gene expression is very interesting‭ (‬Figs‭ ‬5,‭ ‬S2‭) ‬and really speaks to this question.‭*

*‬d‭) ‬In general,‭ ‬the role of stochastic evolutionary processes,‭ ‬and not just selection‭ (‬artificial and natural‭) ‬should be noted.‭ ‬For example,‭ ‬if the AC charr were simply taken from a stock with a different mtDNA haplotype then these differences in the mtDNA genome might not be adaptive,‭ ‬just random. ‭ ‬If the AC fish has much higher mtDNA expression might this be simply a domestication issue and not indicative of selection in SB as stated‭?‬  ‭ ‬Finally,‭ ‬you find that not all mitochondrial transcripts‭ (‬which are transcribed as a polycistronic transcript‭) ‬are found at similar levels‭ (‬Table‭ ‬1‭) – ‬what does this tell you about differential degradation/post-transcriptional processes‭?‬*

*e‭) ‬There is no discussion about the‭ “‬Analyses of polymorphism in Arctic charr transcriptome‭” (‬Table‭ ‬3,‭ ‬4,‭ ‬5‭)‬,‭ ‬except for the mtDNA.*

**Reply:** (‬c,d,e‭) Excellent suggestions. ‬We added in the final discussion section few sentences on domesticated charr vs Benthic/limnetic.‭ ‬Unfortunately we do not have quantitative data on the phenotypes‭ (‬head shape,‭ ‬and jaw‭) ‬of the AC charr and acknowledge that we categorize it as limnetic based on general features.‭

We gladly added a sentence citing neutral forces,‭ ‬and are acutely aware that much of the divergence is likely due to history,‭ ‬drift etc.‭ ‬The domestication can certainly be the driver for the higher expression in AC‭ ‬-‭ ‬but we need transcriptomes from more populations/morphs to address that point.‭ ‬And yes,‭ ‬the variance in RNA levels from different parts of the mtDNA do indeed suggest differential half life of the various RNA species.‭ ‬Some are certainly degraded and others most probably actively utilized‭ ‬/‭ ‬protected.‭ ‬We decided not to follow that thought further though,‭ ‬as the MS already consists of quite a few threads already.

We also added sentences on the genetic polymorphism,‭ ‬before focusing more on the mtDNA.‭ ‬The main reason we dont want to elaborate to much on the SNPs is that we feel these data are mainly for generating hypotheses,‭ ‬and that more work is needed to substantiate SNPs and study their distribution in other populations.

*‭*

*‬Minor Comments‭*

*‬Introduction:‭ ‬*

*a‭) ‬Paragraph‭ ‬3‭ – “‬Furthermore,‭ ‬recent estimates from the rainbow trout‭…‬.by utilizing multiple data sources the genome assembly problem of this family can be solved‭”‬.‭ ‬I am not sure how this statement is relevant to this particular study.‭ ‬This and the following statement seem more appropriate for the methods/discussion to me.*

‬ **Reply:** ‬We deleted this sentence and simplified the paragraph.

‬ *b‭) ‬The morphs being discussed should be clarified throughout the paper.‭ ‬For example,‭ ‬the authors often state‭ “‬among morphs/among charr populations‭” ‬but it is not clear which of the many morphs they are referring to‭ (‬e.g.‭ ‬Paragraph‭ ‬5,‭ ‬first sentence on allozymes and mtDNA and later sentence on MCHIIa‭ – ‬do you mean all‭ ‬4‭ ‬morphs of specific‭ ‬2-way comparisons‭? ‬Are some morphs more differentiated than others‭?)*

**Reply:** ‬We tried to clarify this in various places in the manuscript,‭ ‬but in some cases we refer to morphs in general.‭ ‬Genetic separation can be estimated with Fst values either between pairs or over a larger set of groups‭ (‬populations,‭ ‬morphs‭)‬.‭ ‬In the intro we cite the work done to date in Iceland,‭ ‬which highlights the need for more pop.‭ ‬genetic analyses.‭

‬ ‭

‬ *Methods:‭ ‬*

*a‭) ‬The authors should note why they did not use the PI‭ (‬large piscivorous‭) ‬morph in any qPCR studies‭ (‬in the methods or discussion‭) ‬as this would be a nice morph to use in their tests for parallelism.‭*

**Reply: **The PI charr is very rare in the lake and hard to catch.‭ ‬We later captured few sexually mature individuals,‭ ‬and generated couple of families,‭ ‬that were used for one study‭ (‬Ahi et al‭ ‬Evodevo 2015‭)‬.

*‬b‭) ‬Page‭ ‬5‭ (‬last paragraph‭) – ‬the methods used to remove particular variants needs to be clarified.‭ ‬In particular,‭ ‬why the assumptions used to remove variants are valid by referencing past studies.*

‬ **Reply:** ‬Many of the principles are common to most pipelines for removing spurious variants.‭ ‬In addition we applied filters necessitated by the properties of our dataset‭  (‬pool of individuals‭)‬,‭ ‬the mapping to an outgroup and paralogs due to salmonid genome complexity.

‬ *Figures‭ & ‬Results:‭ ‬*

*a‭) ‬Figure‭ ‬2.‭ ‬The key for Figure‭ ‬2‭ ‬should include a specific heading for morph and time-point with the abbreviations restated‭ [‬e.g.‭ ‬Timepoint:‭ ‬141‭ ‬dpf,‭ ‬Morph:‭ ‬Small Benthic‭ (‬SB‭)]‬.*

**Reply:** ‬Now fixed.‭

‬ ‭

‬ *b‭) ‬Figure‭ ‬6‭ – ‬would be helpful to label the protein coding genes in this figure as well as the‭ ‬12s and‭ ‬16s RNAs.‭*

‬ **Reply:** ‬Now fixed.‭

‬ ‭

‬ *c‭) ‬Figure‭ ‬7‭ – ‬It is not clear to me which variant is present in which morph.‭ ‬Adding the nucleotide to the x-axis‭ (‬i.e.‭ ‬frequency of m1829G for B‭) ‬would make this figure easier to quickly interpret.‭ ‬The‭ “‬A.charr_WT‭” ‬and‭ “‬A.charr_M‭” ‬should also be defined in the legend and it would be more appropriate to use scientific names for all species.‭*

‬ **Reply:** ‬Now fixed‭

‬ *Discussion:‭ ‬*

*a‭) ‬Discussion of reference‭ ‬32‭ – ‬The discussion of reference‭ ‬32‭ ‬is not put into the proper context.‭ ‬Figure‭ ‬6‭ ‬of this paper‭ (‬Berthelot‭ ‬et al.‭ ‬2014‭) ‬shows that there are many genes that have no correlation among expression patterns and/or differences in expression levels‭ (‬1573,‭ ‬1248,‭ ‬and‭ ‬1895‭=‬4716‭ ‬paralog pairs‭)‬,‭ ‬and that together these represent more than the‭ ‬1,407‭ ‬correlated/similar expression level paralogs.‭ ‬This section of the discussion needs to be modified.‭*

**Reply:** ‬Really valuable point,‭ ‬that we are especially grateful for.‭ ‬That we have added this fact to the intro and altered our interpretations in the discussion.

‬ *b‭) ‬The Norman‭ ‬et al.‭ (‬2014‭) ‬paper should be mentioned earlier‭ – ‬if this is available why was it not used for their analyses‭? ‬As well,‭ ‬the last sentence in this paragraph can be cut as it is evident.*

**Reply:** ‬The Norman papers are now presented more clearly in the intro.‭ ‬There are historical reasons for not including their data in our analyses,‭ ‬we had completed the analyses for this manuscript when they became available and have since then focused our data analyses efforts on another transcriptome generated in the lab‭ (‬with longer reads‭)‬.‭

*c‭) ‬Page‭ ‬18‭ – “‬Our new data also demonstrate differences in craniofacial elements between AC-‭ ‬and SB-charr,‭ ‬along a limnetic vs.‭ ‬benthic axis79‭”‬.‭ ‬Are you referring to ref‭ ‬79‭ ‬or data from this study‭? ‬If you are referring to‭ ‬79,‭ ‬clarify and note what you found.‭ ‬This occurs a few times in the discussion‭*

**Reply:** ‬Ref‭ ‬79‭ ‬is a related study that built in part on the data presented here.‭ ‬We have now rephrased this in the manuscript,‭ ‬hopefully to the better.

# Peer review: Referee response for version 1

**DOI:** 10.5256/f1000research.6869.r8970

**Published:** 2015-07-07

**Authors:** Daniel Macqueen

**Affiliations:** 1 Institute of Biological and Environmental Sciences, University of Aberdeen, Aberdeen, UK

**Review of Gudbrandsson *et al*. “The developmental transcriptome of contrasting Arctic charr ( *Salvelinus alpinus*) morphs”.**

The work is founded on the solid premise that rapidly evolving phenotypes in nature can be underpinned by changes at the transcriptome level. The model system here is Arctic charr populations that have evolved (since the last ice age) major differences in phenotypes along the ‘benthic’ - ‘limnetic’ axis, with strong differences in head morphology linked to feeding specializations. The work provides an extensive analysis of transcriptome and genetic differences between different morphs and populations. It is interesting, generally well-written and has merit on many levels. It is also rather hard going, since so much ground is covered on diverse areas. The study also comes with a large number of caveats, of which the authors are undoubtedly aware. Overall though, I am supportive of this work, as it represents one of the most detailed analyses of molecular mechanisms linked to rapid phenotypic evolution in Arctic charr. I see it as a great start point for future work and a source of several new findings and hypotheses. I suggest that the paper be indexed in F1000 Research as long as its caveats are transparent and the authors address my comments.

I list below a number of suggestions that may help the authors improve the work, or that at least highlight study limitations for the benefit of interested readers. I also provide a number of minor comments and suggestions, which should help improve the manuscript more incrementally.

**Main comments & caveats**

1. **RNAseq study design. ** I sympathize with the fact that the authors are trying to publish Illumina data that was generated in 2009, since (obviously) the technology has moved on greatly in the last 6 years, while its costs have been reduced dramatically. Adding to this is the fact that the authors are using a particularly complex transcriptome in terms of high content of similar paralogues (and expressed transposable elements), without a reference sequence for mapping in their species. I accept the author’s argument that it is more sensible to map against a closely related species with the sequence data rather than to try and create a *de novo *assembly from 36bp reads. I also believe it is sensible to pool read counts for putative paralogous contigs in this study, since the short read length ablates any ability to separate paralogous differences in expression (yet does not preclude the generation of useful hypotheses about putative gene expression differences among morphs).
  However, I do question whether the use of Atlantic salmon EST contigs is the best approach here. Firstly, reference assemblies for both Atlantic salmon and rainbow trout are now available, which distinguish paralogous variation. More importantly, using these reference genome data would provide certainty that reads are being mapped to exons from single genes, whereas many of the ESTs will provide a fragmented representation of exon sequences, presumably relying on annotation to piece them back into ‘genes’ *post hoc *. In addition, paired 100bp Ilumina reads are available at high coverage for Arctic charr (e.g. Norman *et al*. 2014), which could also be used to generate a specific reference transcriptome to map against in this study, although this might be underrepresented in terms of developmental genes as it is a gill study. Overall, I do wonder how much more information might have been gleaned from this dataset with a different mapping strategy?
  With all the above said, I understand that the authors have built up a large study based around the original mapping to the salmon ESTs and that it would not be routine for them to repeat the study using better reference data. Furthermore, the approach used has definitely led to the generation of several valid hypotheses concerning the nature of gene expression and genetic differences among charr morphs, which have been followed up using independent approaches.
2. **Methods “Biological Replication in RNAseq” – **a general comment: obviously the design of the study is not optimal because biological variation within developmental stages is not considered in the statistics. Thus, the approach lacks power to detect differences when morph variation is restricted to different developmental stages. I wanted to explain my opinion (for the record) that the study design is nonetheless useful for identifying constitutive differences between morphs. This is especially true because gene expression variability is likely to be relatively low in embryonic stages (compared to a similar study design in adults at least). Further, the pooling of individuals will have helped to at least recapture some biological variation at different stages. Thus, as mentioned above, I see the author’s use of RNAseq as a hypothesis-generating approach, which has been quite fruitful in identifying putative differences between different morphs.
3. **Methods “QPCR study design”. **The authors adhere to the MIQE guidelines, but do not always follow the best approaches. Most pertinently, the authors use the 2 ^−∆∆Ct^ method (assuming PCR efficiency of 2.0) despite having gone to the effort of gaining and reporting efficiencies for each assay, which can be as low as 1.72 for some genes. The effect of failing to incorporate differences in efficiency are highly established and this is likely to have affected the author’s results. The authors should consider incorporating the effect of differences in efficiency into their analyses. This is likely to have some impact on the study conclusions in my opinion.
4. **Methods** “ **Polymorphisms in charr transcriptome”. **While this is not exactly my area of expertise, I struggled to understand the methods behind filtering paralogous variants from SNPs in the data. The authors state “ *As the SNP analysis was done on individual contigs, differences among paralogs appear in the data. However, since each sample is a pool of few individuals, it is very unlikely that we have the same frequency of true SNPs in the samples. This property was used to remove variants that are most likely due to expressed paralogs*”. Can the authors please try to re-explain this in even simpler terms to help me get it? I don’t see how this description leads to a robust identification of paralogous variation. Is there an underlying assumption of equal expression among paralogues? If so, this is likely to be routinely invalidated.
5. **Methods** “ **Verification of candidate SNPs”. **While it is good that the authors have attempted to verify SNPs identified from their RNAseq data, I don’t believe the data is particularly well incorporated in the results section. It needs to be stated up front the extent to which the SNPs predicted from the RNAseq were independently verified. Also, the methods for this section can be improved, especially “ *we conducted genomic comparisons of the Salmon genome, ESTs and short contigs from the preliminary assembly of the Arctic charr transcriptome*”. None of this information is elaborated on – what is the preliminary assembly of the Arctic charr transcriptome? Which version of the salmon genome was used and how? Moreover, it would be useful to actually explain in the methods that the genotyping was done on a small number of SB, PL and PI morphs, rather than relying on the reader to extract all the required information from Table S2. I guess overall, the way this section is incorporated into the manuscript needs some thought in terms of improving the reader’s experience. I struggled after reading it several times and am still not sure I have all the information I need.
6. **Results**. “ *Analyses of those reads require an Arctic charr genome sequence or transcriptome assembly from longer and paired end reads*.” As mentioned already, the latter is available to generate an Arctic charr transcriptome assembly to map against.
7. **Results**; Figure 3 and 4. The authors found that around half the genes studied were not differentially expressed among morphs by qPCR. Obviously this is quite a large number, but on closer inspection, I noticed that *Ndub6*, *Ubl5* and *parp6 *were not even differentially expressed according to RNAseq. Thus, I am confused at the selection of genes from the RNAseq analysis for verification by qPCR. The authors should explain this selection more transparently and provide clearer indices of the correlation between RNAseq and qPCR results and associated discussion.

**Minor comments, typos and suggested changes**

1. Abstract: “ *Species and populations with parallel evolution of specific traits can help illuminate how predictable adaptations and divergence are at the molecular and developmental level*. Grammatically – his reads better: “ *….. can help illuminate the predictability of adaptations and divergence at the molecular and developmental level”*
2. Introduction: “ *Examples of such a species complex are the finches of the Galapagos islands, cichlids in the African great lakes are exciting multi-species systems in this respect”*. Grammatically – reads better: “ *Examples of such species complexes are provided by finches of the Galapagos islands, while cichlids of the African great lakes also provide an exciting multi-species system in the same respect”*
3. Introduction: “ *Some northern freshwater fish species exhibit frequent parallelism in trophic structures and life history and in several cases are they found as distinct resource morphs*” change to “ *…. are found as distinct resource morphs*”
4. Introduction: “ *in the development of ecological differences in tropic morphology*” change to “… *trophic morphology*”.
5. Introduction: “ *The family is estimated to be between 63.2 and 58.1 million years old*”. This information is not correct – it is correct to state that the age of the salmonid crown (based on the cited paper; different estimates exist in the literature, e.g. Macqueen and Johnston, 2014; Campbell *et al*. 2013) is estimated at 63.2 and 58.1 million years old, but the family dates back much further – to the origin of the WGD event in fact, which occurred more like 88-103 Ma (Macqueen and Johnston, 2014; Berthelot *et al*. 2014). Thus, the last common ancestor to extant salmonid species is what the authors are actually referring to in this sentence.
6. Introduction: “ *Furthermore, for data with short reads, mapping to a related reference genome/transcriptome is recommended over de novo assembly*”. While this sentence is technically correct in the context of the work cited, I feel it is being used slightly out of context. For a start, what comprises a ‘short read’ is undefined. 36bp is short, but it is possible to get a sold reference transcriptome using 2*100bp, assuming the appropriate diversity of transcripts is represented and suitable depth is attained.
7. Introduction: “ *nuclear genes, reveled both subtle*” change to “ *nuclear genes, revealed both subtle*”
8. Minor comment – AC, PL, LB and SB were already defined in introduction.
9. Methods: “ *Fishing in Lake Thingvallavatn was with permissions*” changed to “ *Fishing in Lake Thingvallavatn was done with permissions*”.
10. Methods: “ *of differently expressed genes, we preformed clustering analyses*” change to “ *…we performed clustering analyses*”
11. Results: “ *The most drastic changes were seen in processes related to glycolysis (GO:0006096, FDR = 0.0009), were the expression of 19 out of 25 genes*” change to “…. *where the expression*”.
12. Figure 7. What does the charr_WT vs. charr_M signify in the alignment data?
13. Discussion “ *We are interested in how predictable evolution is a the molecular level and if there certain principles influence the rewiring of developmental and regulatory systems during evolution*” consider changing to “ *We are interested in the predictability of evolution at the molecular level, especially whether there exist principles that influence the rewiring of developmental and regulatory systems”*.
14. Discussion. “ *Recent rainbow trout data shows most paralogs from the latest whole genome duplication event retain the same expression pattern ^32^ indicating that this scenario is probably uncommon; hence it is of considerable interest when two paralogs show distinct expression patterns”. *I do not agree that it is of considerable interest when two paralogs show distinct expression patterns – I could list tens of examples for salmonids.
15. Conclusions “ *The results suggest genetic and expression changes in multiple systems relate to divergence among populations*.” Change to “ *… associated with divergence among populations*.”

I have read this submission. I believe that I have an appropriate level of expertise to confirm that it is of an acceptable scientific standard, however I have significant reservations, as outlined above.

# Response

**Published:** 2016-04-04

**Authors:** Arnar Palsson

**Affiliations:** University of Iceland, Iceland

*Main comments‭ & ‬caveats*

*RNAseq study design.‭ ‬ I sympathize with the fact that the authors are trying to publish Illumina data that was generated in‭ ‬2009,‭ ‬since‭ (‬obviously‭) ‬the technology has moved on greatly in the last‭ ‬6‭ ‬years,‭ ‬while its costs have been reduced dramatically.‭ ‬Adding to this is the fact that the authors are using a particularly complex transcriptome in terms of high content of similar paralogues‭ (‬and expressed transposable elements‭)‬,‭ ‬without a reference sequence for mapping in their species.‭ ‬I accept the author’s argument that it is more sensible to map against a closely related species with the sequence data rather than to try and create a‭ ‬de novo‭ ‬assembly from‭ ‬36bp reads.‭ ‬I also believe it is sensible to pool read counts for putative paralogous contigs in this study,‭ ‬since the short read length ablates any ability to separate paralogous differences in expression‭ (‬yet does not preclude the generation of useful hypotheses about putative gene expression differences among morphs‭)‬.‭*

*‬However,‭ ‬I do question whether the use of Atlantic salmon EST contigs is the best approach here.‭ ‬Firstly,‭ ‬reference assemblies for both Atlantic salmon and rainbow trout are now available,‭ ‬which distinguish paralogous variation.‭ ‬More importantly,‭ ‬using these reference genome data would provide certainty that reads are being mapped to exons from single genes,‭ ‬whereas many of the ESTs will provide a fragmented representation of exon sequences,‭ ‬presumably relying on annotation to piece them back into‭ ‘‬genes‭’ ‬post hoc‭ ‬.‭ ‬In addition,‭ ‬paired‭ ‬100bp Ilumina reads are available at high coverage for Arctic charr‭ (‬e.g.‭ ‬Norman‭ ‬et al.‭ ‬2014‭)‬,‭ ‬which could also be used to generate a specific reference transcriptome to map against in this study,‭ ‬although this might be underrepresented in terms of developmental genes as it is a gill study.‭ ‬Overall,‭ ‬I do wonder how much more information might have been gleaned from this dataset with a different mapping strategy‭?*

*With all the above said,‭ ‬I understand that the authors have built up a large study based around the original mapping to the salmon ESTs and that it would not be routine for them to repeat the study using better reference data.‭ ‬Furthermore,‭ ‬the approach used has definitely led to the generation of several valid hypotheses concerning the nature of gene expression and genetic differences among charr morphs,‭ ‬which have been followed up using independent approaches.‭*

‬

**Reply**:‭ ‬We thank the reviewer for excellent diagnosis and suggestions.‭ ‬The paper describes the‭ (‬in our humble opinion‭) ‬most sensible summary of the data,‭ ‬as the writing of the paper started‭ ‬2‭ ‬years ago.‭ ‬We did map on the‭ ‬O.mykiss‭ ‬cDNA collection also,‭ ‬got similar results,‭ ‬but opted for reporting on the salmon data to avoid further extending an already long manuscript.‭ ‬We are currently analyzing DE and SNPs on a new assembly‭ (‬100‭ ‬bp PE reads‭ ‬-‭ ‬48‭ ‬samples‭ ‬-‭ ‬3‭ ‬morphs‭ ‬-‭ ‬development‭)‬,‭ ‬and may include a remapping of this dataset in that.‭

‭    ‬ *Methods‭ “‬Biological Replication in RNAseq‭” – ‬a general comment:‭ ‬obviously the design of the study is not optimal because biological variation within developmental stages is not considered in the statistics.‭ ‬Thus,‭ ‬the approach lacks power to detect differences when morph variation is restricted to different developmental stages.‭ ‬I wanted to explain my opinion‭ (‬for the record‭) ‬that the study design is nonetheless useful for identifying constitutive differences between morphs.‭ ‬This is especially true because gene expression variability is likely to be relatively low in embryonic stages‭ (‬compared to a similar study design in adults at least‭)‬.‭ ‬Further,‭ ‬the pooling of individuals will have helped to at least recapture some biological variation at different stages.‭ ‬Thus,‭ ‬as mentioned above,‭ ‬I see the author’s use of RNAseq as a hypothesis-generating approach,‭ ‬which has been quite fruitful in identifying putative differences between different morphs.*

**‬Reply**:‭ ‬We appreciate the reviewers careful analyses of the study and approach.‭ ‬We tried to emphasize the‭ “‬hypothesis-generation‭” ‬aspect during the rewrite.

*Methods‭ “‬QPCR study design‭”‬.‭ ‬The authors adhere to the MIQE guidelines,‭ ‬but do not always follow the best approaches.‭ ‬Most pertinently,‭ ‬the authors use the‭ ‬2−‭∆∆‬Ct method‭ (‬assuming PCR efficiency of‭ ‬2.0‭) ‬despite having gone to the effort of gaining and reporting efficiencies for each assay,‭ ‬which can be as low as‭ ‬1.72‭ ‬for some genes.‭ ‬The effect of failing to incorporate differences in efficiency are highly established and this is likely to have affected the author’s results.‭ ‬The authors should consider incorporating the effect of differences in efficiency into their analyses.‭ ‬This is likely to have some impact on the study conclusions in my opinion.*

**Reply**: ‬Great point.‭ ‬The qPCR primer efficiencies more than‭ ‬1.90‭ ‬can be easily assumed as‭ ‬2‭ ‬because of the negligible effects.‭ ‬Since we used LinReg software for efficiencies not the traditional method,‭ ‬it takes into account the efficiencies for each test for a given primer pair and discard those have different and lower efficiencies.‭ ‬However,‭ ‬the Natterin-like paralogues were below the cut-off.‭ The statistical analyses were done on deltaCt values, prior to transformation based on efficiencies used for visualization. We ‬now report the graphs of their expression adjusting for the lower efficiency, and state in the results “Note however, the efficiency of the primers for the nattl genes ranged from 1.72 to 1.77, which suggests this data should be interpreted with caution.”‭

‬

*Methods‭ “‬Polymorphisms in charr transcriptome‭”‬.‭ ‬While this is not exactly my area of expertise,‭ ‬I struggled to understand the methods behind filtering paralogous variants from SNPs in the data.‭ ‬The authors state‭ “‬As the SNP analysis was done on individual contigs,‭ ‬differences among paralogs appear in the data.‭ ‬However,‭ ‬since each sample is a pool of few individuals,‭ ‬it is very unlikely that we have the same frequency of true SNPs in the samples.‭ ‬This property was used to remove variants that are most likely due to expressed paralogs‭”‬.‭ ‬Can the authors please try to re-explain this in even simpler terms to help me get it‭? ‬I don’t see how this description leads to a robust identification of paralogous variation.‭ ‬Is there an underlying assumption of equal expression among paralogues‭? ‬If so,‭ ‬this is likely to be routinely invalidated.‭*

‬

**Reply**: ‬We acknowledge this part is a hard read.‭ ‬We rewrote this part of the methods. Here is another summary.‭ ‬Reads from regions that are very similar in paralogous genes can map to both of them.‭ ‬Because we consider also reads that map to many contigs,‭ ‬some of the candidate variants will reflect sequence differences between paralogs,‭ ‬not polymorphism in either paralog.‭ ‬Next we deploy the population genetic argument,‭ ‬since we are sequencing RNA from‭ ‬6‭ ‬chromosomes in each‭  ‬sample,‭ ‬then it is very unlikely that a TRUE SNP will be at the same frequency in all of the‭ ‬8‭ ‬samples.‭ ‬But variants‭ ‬-‭ ‬that are due to differences bwn paralogs‭ ‬-‭ ‬are likely to be similar in frequency because they are unaffected by the population sampling.‭ ‬This filter is designed to toss those out.

To emphasize‭ ‬the objective is not‭ ‬to find differences between paralogs,‭ ‬but rather to enrich for true SNPs.‭ ‬This method will toss out many sites separating paralogous genes (but not all because some paralogous genes are differentially expressed between morphs or time points).‭

*‭    ‬Methods‭ “‬Verification of candidate SNPs‭”‬.‭ ‬While it is good that the authors have attempted to verify SNPs identified from their RNAseq data,‭ ‬I don’t believe the data is particularly well incorporated in the results section.‭ ‬It needs to be stated up front the extent to which the SNPs predicted from the RNAseq were independently verified.‭ ‬Also,‭ ‬the methods for this section can be improved,‭ ‬especially‭ “‬we conducted genomic comparisons of the Salmon genome,‭ ‬ESTs and short contigs from the preliminary assembly of the Arctic charr transcriptome‭”‬.‭ ‬None of this information is elaborated on‭ – ‬what is the preliminary assembly of the Arctic charr transcriptome‭? ‬Which version of the salmon genome was used and how‭? ‬Moreover,‭ ‬it would be useful to actually explain in the methods that the genotyping was done on a small number of SB,‭ ‬PL and PI morphs,‭ ‬rather than relying on the reader to extract all the required information from Table S2.‭ ‬I guess overall,‭ ‬the way this section is incorporated into the manuscript needs some thought in terms of improving the reader’s experience.‭ ‬I struggled after reading it several times and am still not sure I have all the information I need.‭*

**Reply**:‭ ‬We fixed the methods section to accommodate both reviewers which brought up similar points.‭ ‬We highlight the sampling‭ (‬8‭ ‬individuals of‭ ‬3‭ ‬morphs‭)‬,‭ ‬and extend the description of the genomic comparisons.‭ ‬We also extend the discussion of those results.

*Results.‭ “‬Analyses of those reads require an Arctic charr genome sequence or transcriptome assembly from longer and paired end reads.‭” ‬As mentioned already,‭ ‬the latter is available to generate an Arctic charr transcriptome assembly to map against.‭*

‬

**Reply**: ‬Unfortunately the great Norman *et al‭. *‬2014‭ ‬data‭ (‬http://www.ncbi.nlm.nih.gov/pubmed/24368751‭) ‬came to our attention after we had done these analyses,‭ ‬and started working on our new data‭ (‬see above‭)‬.‭ ‬Thus we opted for not redoing the whole analyses for this manuscript,‭ ‬but focus on the verification‭ ‬-‭ ‬and of course working on a new assembly using longer reads.

*Results‭; ‬Figure‭ ‬3‭ ‬and‭ ‬4.‭ ‬The authors found that around half the genes studied were not differentially expressed among morphs by qPCR.‭ ‬Obviously this is quite a large number,‭ ‬but on closer inspection,‭ ‬I noticed that‭ ‬Ndub6,‭ ‬Ubl5‭ ‬and‭ ‬parp6‭ ‬were not even differentially expressed according to RNAseq.‭ ‬Thus,‭ ‬I am confused at the selection of genes from the RNAseq analysis for verification by qPCR.‭ ‬The authors should explain this selection more transparently and provide clearer indices of the correlation between RNAseq and qPCR results and associated discussion.*

**Reply**: ‬This reflects the history of the project,‭ ‬and the difference between the preliminary and final analyses.‭ ‬We decided to report on all the data‭ ‬-‭ ‬but explain better in the manuscript the classification of genes tested with qPCR,‭ ‬at‭ ‬1%,‭ ‬5%‭ ‬and‭ ‬10%‭ ‬FDR.‭ ‬In summary,‭ ‬some of the genes tested were above‭ ‬5%‭ ‬and one even just above‭ ‬10%‭ ‬FDR.‭ ‬Some of those were not corroborated by qPCR.‭ ‬The number of genes is insufficient to do a statistical comparison of the verification rate at the different FDR levels.‭ ‬A table‭ (‬new Table‭ ‬3‭) ‬-‭ ‬supported with few sentences in the results,‭ ‬hopefully clarifies this.

‬ *Minor comments,‭ ‬typos and suggested changes*

*Abstract:‭ “‬Species and populations with parallel evolution of specific traits can help illuminate how predictable adaptations and divergence are at the molecular and developmental level.‭ ‬Grammatically‭ – ‬his reads better:‭ “‬…..‭ ‬can help illuminate the predictability of adaptations and divergence at the molecular and developmental level‭”‬*

**Reply**: ‬Thanks‭ ‬-‭ ‬fixed.

*Introduction:‭ “‬Examples of such a species complex are the finches of the Galapagos islands,‭ ‬cichlids in the African great lakes are exciting multi-species systems in this respect‭”‬.‭ ‬Grammatically‭ – ‬reads better:‭ “‬Examples of such species complexes are provided by finches of the Galapagos islands,‭ ‬while cichlids of the African great lakes also provide an exciting multi-species system in the same respect‭”‬*

**Reply**:‭ ‬Thanks‭ ‬-‭ ‬fixed.

*Introduction:‭ “‬Some northern freshwater fish species exhibit frequent parallelism in trophic structures and life history and in several cases are they found as distinct resource morphs‭” ‬change to‭ “‬….‭ ‬are found as distinct resource morphs‭”‬*

**Reply**: ‬Thanks‭ ‬-‭ ‬fixed.

*Introduction:‭ “‬in the development of ecological differences in tropic morphology‭” ‬change to‭ “… ‬trophic morphology‭”‬.‭*

‬ **Reply**: ‬Thanks‭ ‬-‭ ‬fixed.

*Introduction:‭ “‬The family is estimated to be between‭ ‬63.2‭ ‬and‭ ‬58.1‭ ‬million years old‭”‬.‭ ‬This information is not correct‭ – ‬it is correct to state that the age of the salmonid crown‭ (‬based on the cited paper‭; ‬different estimates exist in the literature,‭ ‬e.g.‭ ‬Macqueen and Johnston,‭ ‬2014‭; ‬Campbell‭ ‬et al.‭ ‬2013‭) ‬is estimated at‭ ‬63.2‭ ‬and‭ ‬58.1‭ ‬million years old,‭ ‬but the family dates back much further‭ – ‬to the origin of the WGD event in fact,‭ ‬which occurred more like‭ ‬88-103‭ ‬Ma‭ (‬Macqueen and Johnston,‭ ‬2014‭; ‬Berthelot‭ ‬et al.‭ ‬2014‭)‬.‭ ‬Thus,‭ ‬the last common ancestor to extant salmonid species is what the authors are actually referring to in this sentence.*

**Reply**: ‬Thanks so for pointing this out.‭ ‬We changed the text to‭ “‬local adaptation has been extensively studied in the salmonid family,‭ ‬to which Arctic charr belongs‭ {‬Fraser2011‭}‬.‭ ‬The family is estimated to be between‭ ‬88-103‭ ‬million years old‭ {‬Macqueen2014,Berthelot2014c‭}‬.‭ ‬A whole genome duplication event occurred before the radiation of the salmonid family‭ {‬Davidson2010,Moghadam2011,Macqueen2014,Berthelot2014c‭} ‬which has provided time for divergence of ohnologous genes‭ (‬paralogous genes originated by whole genome duplication event‭)‬.‭ ” ‬

Introduction:‭ “‬Furthermore,‭ ‬for data with short reads,‭ ‬mapping to a related reference genome/transcriptome is recommended over de novo assembly‭”‬.‭ ‬While this sentence is technically correct in the context of the work cited,‭ ‬I feel it is being used slightly out of context.‭ ‬For a start,‭ ‬what comprises a‭ ‘‬short read‭’ ‬is undefined.‭ ‬36bp is short,‭ ‬but it is possible to get a sold reference transcriptome using‭ ‬2‭*‬100bp,‭ ‬assuming the appropriate diversity of transcripts is represented and suitable depth is attained.‭

‬

**Reply**: ‬Great point,‭ ‬we opted for keeping the point‭ (‬at this place in the ms‭) ‬but changing the wording to:‭ ‬In this study we opted to map the reads‭ (‬36‭ ‬bp‭) ‬to a related reference genome/transcriptome‭ {‬Vijay2013a‭}‬,‭ ‬instead of conducting de novo assembly.‭

*Introduction:‭ “‬nuclear genes,‭ ‬reveled both subtle‭” ‬change to‭ “‬nuclear genes,‭ ‬revealed both subtle‭”*

**Reply**: ‬Thanks fixed.‭

‬

*Minor comment‭ – ‬AC,‭ ‬PL,‭ ‬LB and SB were already defined in introduction.‭*

‬ **Reply**: ‬Thanks, removed this.

*Methods:‭ “‬Fishing in Lake Thingvallavatn was with permissions‭” ‬changed to‭ “‬Fishing in Lake Thingvallavatn was done with permissions‭”‬.‭*

‬ **Reply**: Ammended.

*Methods:‭ “‬of differently expressed genes,‭ ‬we preformed clustering analyses‭” ‬change to‭ “‬…we performed clustering analyses‭”‬*

**Reply**: ‬Thanks,‭ ‬fixed.

*Results:‭ “‬The most drastic changes were seen in processes related to glycolysis‭ (‬GO:0006096,‭ ‬FDR‭ = ‬0.0009‭)‬,‭ ‬were the expression of‭ ‬19‭ ‬out of‭ ‬25‭ ‬genes‭” ‬change to‭ “…‬.‭ ‬where the expression‭”‬.‭*

‬ **Reply**:‭ ‬Thanks,‭ ‬fixed.

*Figure‭ ‬7.‭ ‬What does the charr_WT vs.‭ ‬charr_M signify in the alignment data‭?‬*

**Reply**: ‬Designates the two alleles,‭ ‬the legend now makes this explicit.‭

‭ *‬Discussion‭ “‬We are interested in how predictable evolution is a the molecular level and if there certain principles influence the rewiring of developmental and regulatory systems during evolution‭” ‬consider changing to‭ “‬We are interested in the predictability of evolution at the molecular level,‭ ‬especially whether there exist principles that influence the rewiring of developmental and regulatory systems‭”‬.‭*

‬ **Reply**: ‬Thanks,‭ ‬excellent suggestion,‭ ‬included

*Discussion.‭ “‬Recent rainbow trout data shows most paralogs from the latest whole genome duplication event retain the same expression pattern32‭ ‬indicating that this scenario is probably uncommon‭; ‬hence it is of considerable interest when two paralogs show distinct expression patterns‭”‬.‭ ‬I do not agree that it is of considerable interest when two paralogs show distinct expression patterns‭ – ‬I could list tens of examples for salmonids.‭*

‬ **Reply**:‭ ‬Good point,‭ ‬we have revisited this interpretation‭ (‬see also point by rev.‭ ‬1‭)‬.‭

‭    ‬ *Conclusions‭ “‬The results suggest genetic and expression changes in multiple systems relate to divergence among populations.‭” ‬Change to‭ “‬… associated with divergence among populations.‭”*

**Reply**: ‬Thanks,‭ ‬fixed.
